# Astragaloside IV as a promising therapeutic agent for liver diseases: current landscape and future perspectives

**DOI:** 10.3389/fphar.2025.1574154

**Published:** 2025-04-23

**Authors:** Chunyan Chen, Xiaolan Bu, Liping Deng, Jiayan Xia, Xinming Wang, Li Chen, Wen Li, Jie Huang, Qixiang Chen, Cheng Wang

**Affiliations:** ^1^ School of Clinical Medical, Chengdu Medical College, Chengdu, China; ^2^ Department of Pharmacy, The First Affiliated Hospital of Chengdu Medical College, Chengdu, China; ^3^ Department of Otolaryngology-Head and Neck Surgery, The First Affiliated Hospital of Chengdu Medical College, Chengdu, China; ^4^ Department of Orthopedics, The First Affiliated Hospital of Chengdu Medical College, Chengdu, China; ^5^ Department of Pediatrics, The First Affiliated Hospital of Chengdu Medical College, Chengdu, China; ^6^ Department of Respiratory and Critical Care Medicine, The First Affiliated Hospital of Chengdu Medical College, Chengdu, China

**Keywords:** Astragaloside IV, hepatoprotection, liver injury, metabolic-associated fatty liver disease, liver fibrosis, liver cancer

## Abstract

Astragaloside IV (C_41_H_68_O_14_, AS-IV) is a naturally occurring saponin isolated from the root of *Astragalus membranaceus*, a widely used traditional Chinese botanical drug in medicine. In recent years, AS-IV has attracted considerable attention for its hepatoprotective properties, which are attributed to its low toxicity as well as its anti-inflammatory, antioxidant and antitumour effects. Numerous preclinical studies have demonstrated its potential in the prevention and treatment of various liver diseases, including multifactorial liver injury, metabolic-associated fatty liver disease, liver fibrosis and liver cancer. Given the promising hepatoprotective potential of AS-IV and the growing interest in its research, this review provides a comprehensive summary of the current state of research on the hepatoprotective effects of AS-IV, based on literature available in databases such as CNKI, PubMed, ScienceDirect, Google Scholar and Web of Science. The hepatoprotective mechanisms of AS-IV are multifaceted, encompassing the inhibition of inflammatory responses, reduction of oxidative stress, improvement of insulin and leptin resistance, modulation of the gut microbiota, suppression of hepatocellular carcinoma cell proliferation and induction of tumour cell apoptosis. Notably, key molecular pathways involved in these effects include Nrf2/HO-1, NF-κB, NLRP3/Caspase-1, JNK/c-Jun/AP-1, PPARα/FSP1 and Akt/GSK-3β/β-catenin. Toxicity studies indicate that AS-IV has a high level of safety. In addition, this review discusses the sources, physicochemical properties, and current challenges in the development and clinical application of AS-IV, providing valuable insights into its potential as a hepatoprotective agent in the pharmaceutical and nutraceutical industries.

## Graphical Abstract

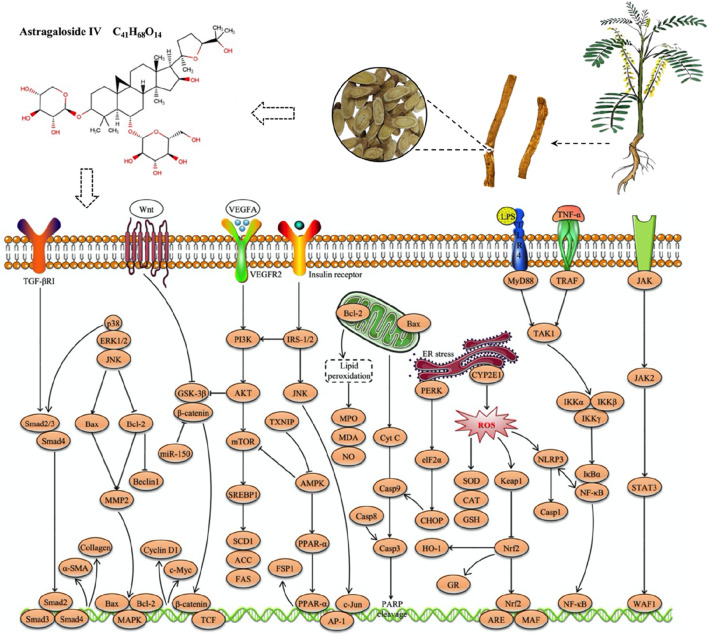

## 1 Introduction

The liver is the largest solid organ in the human body and is responsible for critical functions such as metabolic regulation, detoxification, bile secretion and immune defence, playing a central role in maintaining homeostasis (Kubes and Jenne, 2018; Zaefarian et al., 2019). However, the liver is highly susceptible to damage from external toxins, metabolic disorders and viral infections, leading to acute or chronic liver injury (Arroyo et al., 2020; Taru et al., 2024). Currently, liver diseases, including various forms of liver injury, metabolic-associated fatty liver disease (MAFLD), liver fibrosis and hepatocellular carcinoma (HCC), are an increasingly serious global public health problem with rising prevalence rates that pose a significant threat to patients’ quality of life and survival (Kleinert and Horton, 2022; Karlsen et al., 2024). Although existing treatments show some efficacy in the treatment of certain liver diseases, drug therapy remains limited by suboptimal outcomes and significant side effects (Neshat et al., 2021). Therefore, there is an urgent need to develop safer and more effective therapeutic strategies.

Natural products have become a focus of research in drug development and disease prevention due to their structural diversity, significant biological activities and relatively high safety profile (Meng et al., 2018; Yan et al., 2020). Astragaloside IV (AS-IV), one of the major active metabolites isolated from the traditional Chinese medicine *Astragalus membranaceus*, belongs to the family of triterpenoid saponins and has attracted considerable attention for its diverse biological effects (Zhang et al., 2020). Recent studies have shown that AS-IV possesses multiple pharmacological activities, including antioxidant, anti-inflammatory and antitumour effects, which show promising protective benefits in the prevention and treatment of various chronic diseases (Liang et al., 2023; Xia et al., 2023). In particular, in the field of liver protection, AS-IV shows therapeutic potential through various mechanisms such as inhibition of inflammatory responses, reduction of oxidative stress, amelioration of insulin and leptin resistance, modulation of gut microbiota, suppression of HCC cell proliferation and induction of tumour cell apoptosis (Li L. et al., 2018; Li Y. et al., 2018; Su et al., 2020; Zhou et al., 2021; Zhai et al., 2022). More importantly, emerging evidence suggests that several key signaling pathways, including nuclear factor erythroid 2-related factor 2 (Nrf2)/heme oxygenase 1 (HO-1), nuclear factor kappa-B (NF-κB), NOD-like receptor family pyrin domain containing 3 (NLRP3)/Caspase-1, c-Jun N-terminal kinase (JNK)/c-Jun/activator protein 1 (AP-1), peroxisome proliferator-activated receptor alpha (PPARα)/fibroblast specific protein 1 (FSP1), and protein kinase B (Akt)/glycogen synthase kinase 3β (GSK-3β)/β-catenin, play a critical role in AS-IV-mediated liver protection (Cheng et al., 2011; Wang P. P. et al., 2017; Qu et al., 2019; Li L. et al., 2022; Wu et al., 2023).

Given the growing interest and therapeutic potential of AS-IV in liver protection, this review systematically summarizes its pharmacological effects and underlying mechanisms in liver diseases based on literature retrieved from databases such as CNKI, PubMed, ScienceDirect, Google Scholar and Web of Science. Furthermore, we analyze the limitations of current studies and suggest important future research directions to provide a more comprehensive and scientific basis for the clinical application of AS-IV.

## 2 Sources and characteristics of AS-IV

**FIGURE 1:**
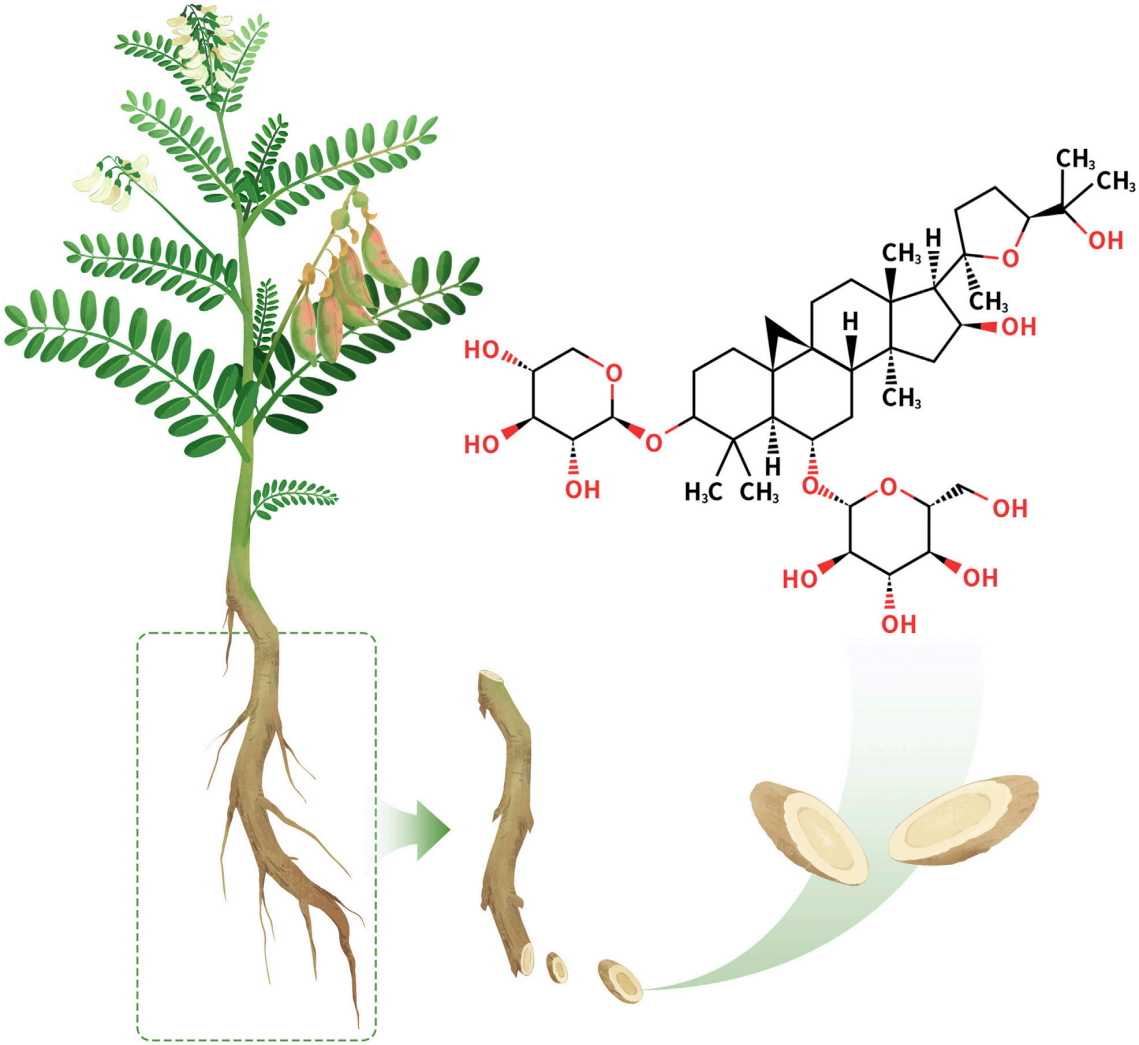
Chemical structure of AS-IV.

AS-IV (Figure 1) is a triterpenoid saponin metabolite isolated from the traditional Chinese medicine *Astragali Radix* with the molecular formula C_41_H_68_O_14_ (Zhang et al., 2020). AS-IV appears as a white crystalline powder with well-defined physicochemical properties (CAS: 83207-58-3; UNII: 3A592W8XKE). It exhibits unique solubility characteristics, with a solubility of 30 mg/mL in DMSO, which decreases significantly to 0.5 mg/mL in PBS buffer, suggesting the need for solubilization strategies during formulation development. In addition, its LogP value of 1.298 ± 0.00 (Temp: 25°C) indicates moderate lipophilicity and hydrophilicity, providing a potential advantage for membrane permeability and drug delivery.

However, the low solubility of AS-IV limits its bioavailability, thereby affecting its clinical efficacy. To address this, several strategies have been proposed to improve the solubility and bioavailability of AS-IV. For example, the use of nanocarriers, such as nanoparticles, can significantly enhance the solubility of AS-IV and increase its absorption in the body (Zhao et al., 2023; Qu et al., 2025). Additionally, prodrug strategies have also been recognized as effective methods, where chemical modifications of AS-IV improve its solubility and convert it into its active form within the body to enhance its therapeutic effect (Li Z. et al., 2022; Wu et al., 2022). Liposome formulations can encapsulate AS-IV, not only enhancing its stability but also improving its targeted delivery, thereby further increasing its bioavailability (Kuo et al., 2021).

In recent years, exosomes have emerged as a novel drug delivery system that is attracting increasing attention. Exosomes can effectively protect drug molecules from rapid degradation in the body and facilitate targeted drug delivery through paracellular transport (Cheng and Hill, 2022). For AS-IV, exosome carriers not only improve its solubility, but also enhance its targeting ability, optimize its distribution in the body and significantly increase its bioavailability (Shan et al., 2024; Yang et al., 2024). These innovative strategies have further expanded the clinical application potential of AS-IV.

In terms of chemical stability, AS-IV has a melting point range of 284°C–286°C and a remarkably high boiling point of 895.666°C ± 65.00°C, together with an extremely low vapor pressure and a high flash point of 495.481°C ± 34.28°C, indicating its excellent thermal stability and low volatility. The molecular structure of AS-IV contains multiple hydroxyl and glycosyl groups, which enable it to interact with various biomolecules through hydrogen bonding and hydrophobic interactions, thereby imparting notable antioxidant, anti-inflammatory, and immunomodulatory activities.

In summary, AS-IV, with its stable physicochemical properties and diverse pharmacological activities, has emerged as a key candidate for the modernization of traditional Chinese medicine and the development of innovative drugs. Strategies to enhance its bioavailability, including nanocarriers, prodrugs, liposomal formulations, and exosome-based delivery systems, may offer broader prospects for the clinical application of AS-IV. Table 1 presents comprehensive data on the physicochemical properties of AS-IV, sourced from the PubChem, SciFinder, and ChemSpider.

**TABLE 1:** Physical and chemical properties of AS-IV.

Name	Astragaloside IV
Source	*Astragali Radix*
CAS number	83207-58-3
PubChem CID	168314659
ChemSpider ID	32702087
UNII number	3A592W8XKE
Molecular formula	C_4_1H_68_O_14_
Molecular weight	784.97
Form	Powder
Color	White
Solubility	DMF: 20 mg/mL; DMSO: 30 mg/mL DMSO: PBS (pH 7.2) (1:1): 0.5 mg/mL
InChIKey	QMNWISYXSJWHRY-AUJDEUPOSA-N
Density	1.395 ± 0.10 g/cm^3^ (Temp: 25°C; Press: 760 Torr)
pKa	12.907 ± 0.70 (Most Acidic Temp: 25°C)
Boiling point	895.666°C ± 65.00°C (Press: 760.00 Torr)
Melting point	284°C–286°C
Flash point	495.481°C ± 34.28°C
Vapor pressure	0.00 ± 0.63 Torr (Temp: 25.00°C)
Index of refraction	1.621
Polar surface area	228 Å^2^
LogP	1.298 ± 0.00 (Temp: 25°C)
Storage conditions	Protected from light, 2°C–8°C

## 3 The hepatoprotective effects and mechanisms of AS-IV in liver injury

In the cascade of events leading to the progression of liver disease, acute liver injury serves as both the initiating step for various chronic liver diseases and a key window for evaluating the protective effects of drugs (Zhang et al., 2024). In recent years, the active metabolite AS-IV from traditional Chinese medicine has garnered attention due to its unique dual-modulatory characteristics of “damage repair and homeostasis reconstruction”. Studies have shown that AS-IV not only enhances the antioxidant defense system by activating the Nrf2 pathway, but also precisely regulates Kupffer cells, blocking the spread of inflammatory cytokine storms to surrounding tissues. This dual-intervention strategy targeting oxidative stress and inflammation in the early stages of injury lays a molecular foundation for exploring the multidimensional regulatory mechanisms of AS-IV in more complex liver disease networks.

### 3.1 Drug-induced liver injury

#### 3.1.1 Cisplatin

Cisplatin is a widely used broad-spectrum anticancer agent for the treatment of various malignancies (Rocha et al., 2018). Its anticancer mechanism involves binding to DNA and inhibiting tumour cell proliferation (Rocha et al., 2018). However, while exerting its antitumour effects, cisplatin also induces a number of adverse effects, of which hepatotoxicity is relatively common. Multiple mechanisms are involved in cisplatin-induced liver injury, including oxidative stress, inflammatory responses, mitochondrial dysfunction and apoptosis (Abd Rashid et al., 2021). These mechanisms act synergistically and ultimately lead to liver dysfunction characterized by elevated serum transaminase levels, hepatocyte necrosis and inflammatory infiltration (Oliveira et al., 2024). Fortunately, studies using cisplatin-induced liver injury models in mice and rats have shown that AS-IV significantly alleviates liver injury, inflammation and oxidative stress (Qu et al., 2019; Guo et al., 2024). On the one hand, AS-IV attenuates cisplatin-induced liver injury by inhibiting ferroptosis through the PPARα/FSP1 signaling pathway (Qu et al., 2019). On the other hand, it protects against cisplatin-induced liver and kidney injury *via* autophagy-mediated suppression of the NLRP3 inflammasome (Guo et al., 2024). These findings suggest that AS-IV is a promising potential phytotherapeutic agent for the prevention and treatment of cisplatin-induced liver injury.

#### 3.1.2 Acetaminophen

Acetaminophen (APAP) is a common antipyretic and analgesic, widely prescribed to relieve headache, muscle pain and fever. However, an overdose of paracetamol can cause acute liver damage and even acute liver failure (Jaeschke and Ramachandran, 2024). In fact, APAP-induced liver injury is a multifactorial process involving glutathione (GSH) depletion, oxidative stress, mitochondrial damage, inflammatory response, autophagy inhibition and ferroptosis (Yan et al., 2018; Tan et al., 2020). Therefore, there is an urgent need to identify effective antidotes to mitigate APAP-induced acute liver injury. Interestingly, Li L. et al., 2018) showed that AS-IV (20, 40 mg/kg) provided significant protection against APAP-induced hepatotoxicity. The protective effects were demonstrated by a reduction in serum alanine aminotransferase (ALT) and aspartate aminotransferase (AST) levels, as well as increased hepatic GSH and superoxide dismutase (SOD) activity (Li L. et al., 2018). In addition, AS-IV reversed the elevation of the pro-inflammatory cytokines interleukin-1β (IL-1β), IL-6 and tumor necrosis factor α (TNF-α) in liver tissue (Li L. et al., 2018). Excessive production of pro-inflammatory cytokines has been reported to be an important precursor of APAP-induced liver injury (Jaeschke and Ramachandran, 2024). The hepatoprotective effects of AS-IV are likely related to its potent antioxidant and anti-inflammatory properties, particularly through activation of the Nrf2 pathway, which attenuates oxidative stress and inflammation (Li L. et al., 2018). These findings provide new insights into the potential use of AS-IV in the treatment of APAP-induced liver injury.

#### 3.1.3 Streptozotocin

Streptozotocin (STZ) is commonly used to induce diabetic animal models due to its selective destruction of pancreatic β-cells (Ghasemi et al., 2014). However, STZ also causes some degree of hepatotoxicity, with the mechanism of liver injury closely linked to oxidative stress, inflammatory responses and mitochondrial dysfunction (Massart et al., 2021). Interestingly, AS-IV exhibits significant hepatoprotective effects against STZ-induced diabetic liver injury (Xu, 2017; Zhu, 2021). Specifically, AS-IV effectively reduces fasting blood glucose, fasting serum insulin, triglycerides, total cholesterol, ALT and AST levels in diabetic rats (Xu, 2017; Zhu, 2021). It also enhances insulin sensitivity and corrects dyslipidaemia, while alleviating hepatic oxidative stress, inflammation, and apoptosis (Xu, 2017; Zhu, 2021). Mechanistically, AS-IV improves insulin sensitivity and restores impaired autophagy by modulating phosphatidylinositide 3-kinase (PI3K)/AKT and AMP-activated protein kinase (AMPK)/mammalian target of rapamycin (mTOR) signaling pathways, thereby protecting against liver injury in diabetic rats (Xu, 2017; Zhu, 2021). These findings highlight the potential application of AS-IV in the treatment of diabetes-associated liver injury.

#### 3.1.4 Cyclophosphamide

Cyclophosphamide (CPA) is an alkylating agent widely used in the treatment of cancer and immune disorders. However, the active metabolites formed during its metabolism exhibit significant toxicity, particularly to the liver (Emadi et al., 2009). Among these, acrolein is considered to be the primary metabolite responsible for liver damage, as it directly interacts with cellular proteins, lipids, and nucleic acids, disrupting hepatocyte structure and function (Üsküdar Cansu et al., 2019). Cao et al. (2023) demonstrated that AS-IV (0.1, 0.2, 0.5 g/kg) not only improved the growth performance of tilapia in a CPA-induced injury model, but also significantly enhanced their antioxidant capacity and immune function. Specifically, AS-IV increased final body weight, relative weight gain and specific growth rate, while increasing the activity of peripheral blood leukocytes and head kidney macrophages (Cao et al., 2023). It also upregulated the expression of antioxidant-related genes and immune-related genes (Cao et al., 2023). This protective effect may be attributed to the ability of AS-IV to scavenge free radicals, reduce lipid peroxidation and stabilize immune cell populations (Cao et al., 2023). These results suggest that AS-IV has great potential as a feed additive in aquaculture to improve growth performance and health status.

### 3.2 Chemical liver injury

#### 3.2.1 Alcohols

Chronic or excessive alcohol consumption induces both direct and indirect liver injury through multiple mechanisms, including oxidative stress, dyslipidaemia, inflammation, cell apoptosis, and fibrosis (Lackner and Tiniakos, 2019; Chen M. et al., 2023). Among these, oxidative stress is one of the key factors in ethanol-induced liver injury. Reactive oxygen species (ROS) and reactive nitrogen species generated during ethanol metabolism damage biomolecules such as lipids, proteins and nucleic acids, leading to hepatocyte apoptosis and necrosis (Wu and Cederbaum, 2009). In addition, ethanol inhibits the activity of the endogenous antioxidant system, further weakening the liver’s ability to scavenge oxidants and increasing hepatocyte susceptibility to oxidative stress (Wu and Cederbaum, 2009). Interestingly, Han et al. (2014) showed that AS-IV (0.1, 0.5, 1, 5 mg/L) could reverse the decrease in antioxidant enzyme activities (T-AOC, SOD, CAT, GSH-Px) induced by ethanol in Chang liver cells and significantly reduce the elevated levels of ALT, AST and malondialdehyde (MDA). Furthermore, AS-IV alleviated ethanol-induced G0/G1 phase arrest and had some inhibitory effect on ethanol-induced cell apoptosis (Han et al., 2014).

Acetaldehyde produced during ethanol metabolism and oxidative stress can activate Kupffer cells in the liver and induce the release of pro-inflammatory cytokines such as TNF-α, IL-1β and IL-6, exacerbating the inflammatory response of the liver (Barnes et al., 2014). In addition, this inflammatory response leads to the infiltration of neutrophils and macrophages, further exacerbating hepatocyte damage (Barnes et al., 2014). Similarly, in a mouse model of ethanol-induced liver injury, AS-IV (50, 150, 500 mg/kg) demonstrated significant hepatoprotective effects, as evidenced by a marked reduction in the expression of TNF-α, IL-1β, IL-6, lipopolysaccharide (LPS), LPS-binding protein (LBP), diamine oxidase (DAO) and myeloperoxidase (MPO) in serum, and a significant increase in liver levels of SOD and GSH-Px (Wu et al., 2023). Furthermore, 16S rRNA high-throughput sequencing results showed that AS-IV could improve the gut microbiota imbalance and restore the abundance of beneficial bacterial species such as *Turicibacter*, *Butyricicoccus* and *Akkermansia* to normal levels (Wu et al., 2023). Mechanistically, AS-IV may alleviate liver oxidative stress and inflammation by modulating the composition of the gut microbiota and the NLRP3/Caspase-1 signaling pathway, thereby ameliorating ethanol-induced liver injury in mice (Wu et al., 2023).

#### 3.2.2 Carbon tetrachloride

The carbon tetrachloride (CCl_4_)-induced liver injury model is widely used to study the pathological mechanisms of drug-induced liver injury and potential therapeutic strategies, as it closely resembles the pathological process of human chronic liver disease (Scholten et al., 2015). In brief, CCl_4_ is metabolized in the liver by the cytochrome P450 enzyme system to produce highly reactive free radicals, CCl_3_-and CCl_3_OO- (Unsal et al., 2020). These free radicals induce hepatocyte damage through oxidative stress, lipid peroxidation, inflammatory responses and apoptosis (Weber et al., 2003). In models of CCl_4_-induced liver injury in mice and rats, AS-IV (20–160 mg/kg) showed significant hepatoprotective effects as evidenced by a reduction in ALT, AST, MDA, IL-6, TNF-α and TBIL levels and a significant increase in SOD, GSH and ALB activity (Jiang et al., 2015; Xu and Li, 2019). In addition, AS-IV significantly downregulated the expression of TGF-β and mothers against decapentaplegic homolog 3 (Smad3) proteins in liver tissue, suggesting that it may ameliorate liver injury by inhibiting the TGF-β/Smad signaling pathway (Jiang et al., 2015). These findings suggest that AS-IV has the potential to alleviate CCl_4_-induced oxidative stress and inflammation, providing new insights into the treatment of drug-induced liver injury.

#### 3.2.3 Iron overload

Iron overload-induced liver injury results from excessive iron deposition in the liver, leading to a range of pathological changes (Armstrong et al., 2022). As the primary iron storage organ in the body, the liver is particularly susceptible to iron overload. Excess iron generates a significant number of free radicals *via* the Fenton reaction, particularly hydroxyl radicals and superoxide anions, which induce oxidative stress and lipid peroxidation, ultimately disrupting cell membrane integrity (Ramm and Ruddell, 2010). Xie et al. (2019) showed that in an iron dextran-induced LO2 cell model, AS-IV (10, 20, 40 μM) could reverse the decrease in cell viability caused by iron overload and reduce the apoptosis rate. Transmission electron microscopy revealed a significant increase in autophagosomes after iron dextran treatment, whereas AS-IV treatment significantly reduced the number of autophagosomes (Xie et al., 2019). Furthermore, AS-IV dose-dependently upregulated hepcidin levels, suggesting a regulatory role in iron metabolism (Xie et al., 2019). The hepatoprotective effect of AS-IV against iron overload-induced liver injury may be partly due to its inhibition of excessive autophagy and reduction of apoptosis (Xie et al., 2019). However, the underlying mechanisms of this protective effect need to be further investigated in future studies.

#### 3.2.4 Sodium taurocholate

Acute pancreatitis is a sudden inflammatory condition of the pancreas, primarily characterized by pancreatic tissue oedema, inflammatory response, and enzyme-mediated cellular necrosis (Mederos et al., 2021). Clinically, patients typically present with acute upper abdominal pain, nausea, vomiting, and markedly elevated serum amylase levels (Lee and Papachristou, 2019). Unfortunately, a wide range of inflammatory mediators are released during an episode of acute pancreatitis, often resulting in multi-organ damage, particularly liver injury (Szatmary et al., 2022). Hence, monitoring and protecting liver function during the diagnosis and treatment of acute pancreatitis is critical to improving patient prognosis. In a rat model of acute pancreatitis-induced liver injury induced by taurocholic acid sodium salt, AS-IV (20 mg/kg) significantly reduced serum levels of amylase, ALT, AST and the pro-inflammatory cytokines IL-6, TNF-α and IL-1β. AS-IV also inhibited the phosphorylation of Janus kinase 2 (JAK2) and signal transducer and activator of transcription 3 (STAT3) in liver tissue (Zhang and Chang, 2016). The hepatoprotective effect of AS-IV in acute pancreatitis-associated liver injury may be mediated through modulation of the JAK2/STAT3 pathway (Zhang and Chang, 2016). This provides experimental evidence and theoretical support for the use of AS-IV to protect the liver in acute pancreatitis.

### 3.3 Immune-mediated liver injury

LPS is a major component of the outer membrane of Gram-negative bacteria and its induced liver injury model is widely used to study liver inflammation and immune-mediated mechanisms of liver injury (Pfalzgraff and Weindl, 2019). LPS-induced liver injury occurs primarily through the activation of the liver’s innate immune system, particularly Kupffer cells, leading to the release of large amounts of inflammatory cytokines such as IL-1β, IL-6 and TNF-α (Rathinam et al., 2019). These cytokines not only promote further recruitment of immune cells, but also exacerbate local inflammation, leading to hepatocyte damage and destruction of liver tissue (Rathinam et al., 2019). In a mouse model of LPS-induced liver injury, AS-IV (10 mg/kg) significantly inhibited serum levels of monocyte chemoattractant protein-1 (MCP-1) and TNF-α, reducing them by 82% and 49% respectively (Zhang and Frei, 2015). AS-IV also suppressed the upregulation of inflammatory genes LPS is a major component of the outer membrane of Gram-negative bacteria, and its induced liver injury model is widely used to study liver inflammation and immune-mediated mechanisms of liver injury (Pfalzgraff and Weindl, 2019). LPS-induced liver injury occurs primarily through the activation of the liver’s innate immune system, particularly Kupffer cells, leading to the release of large amounts of inflammatory cytokines such as IL-1β, IL-6 and TNF-α (Rathinam et al., 2019). These cytokines not only promote further recruitment of immune cells, but also exacerbate local inflammation, leading to hepatocyte damage and destruction of liver tissue (Rathinam et al., 2019). In a mouse model of LPS-induced liver injury, AS-IV (10 mg/kg) significantly inhibited serum levels of monocyte chemoattractant protein-1 (MCP-1) and TNF-α, reducing them by 82% and 49%, respectively (Zhang and Frei, 2015). AS-IV also suppressed the upregulation of inflammatory genes in multiple organs, including the liver, lung, heart, aorta and kidney (Zhang and Frei, 2015). Mechanistically, AS-IV inhibited the LPS-induced acute inflammatory response by modulating the NF-κB and AP-1 pathways (Zhang and Frei, 2015).

D-galactosamine (D-GalN) is an amino sugar substance that induces hepatocyte apoptosis and necrosis by specifically interfering with UDP-galactose synthesis and ribosomal RNA transcription in liver cells (Maes et al., 2016). However, D-GalN alone does not cause significant liver injury; it only exerts significant hepatotoxicity when combined with LPS (Kim et al., 2014). Thus, the D-GalN and LPS-induced liver injury model is widely used to study acute liver injury and immune-mediated mechanisms of liver injury (Farghali et al., 2016; Tao et al., 2022). Notably, this model induces severe liver injury by simulating bacterial endotoxin exposure and hepatic metabolic dysfunction, and can lead to pathological features similar to human fulminant liver failure. Liu (2014) demonstrated *in vivo* that AS-IV (50, 150, 500 mg/kg) significantly protected against D-GalN/LPS-induced acute liver failure in mice. Mechanistically, AS-IV may increase the expression of the antioxidant protein HO-1 through modulation of the Nrf2 signaling pathway, thereby alleviating oxidative stress-induced acute liver injury (Liu, 2014). Furthermore, AS-IV may inhibit hepatocyte apoptosis by upregulating the expression of the anti-apoptotic protein B-cell lymphoma-2 (Bcl-2) and reducing the levels of Bcl-2-associated X (Bax) and Caspase-3, further contributing to its protective effects (Liu, 2014).

*Bacillus* Calmette-Guérin (BCG) is an attenuated strain of *Mycobacterium* bovis that is commonly used as an activator of hepatic macrophages in liver injury research (Chen J. et al., 2023). More importantly, the combination of BCG and LPS can be used to establish an infection-induced immune-mediated liver injury model. In this model, BCG acts as an initial activator, sensitizing macrophages and Kupffer cells, while LPS acts as a subsequent inducer, triggering a robust immune response that leads to the significant release of inflammatory factors and exacerbates liver inflammation and injury (Samuel et al., 2024; Wang et al., 2024). Interestingly, in the BCG/LPS-induced liver injury rat model, AS-IV (20, 40, 80 mg/kg) not only reduced organ indices and serum AST and ALT activities, but also reversed the levels of TNF-α, IL-6, MDA and SOD (Gong and Wang, 2014). These findings suggest that AS-IV may have potential protective effects against immune-mediated liver injury, and its mechanism of action may be closely related to its anti-inflammatory and antioxidant activities (Gong and Wang, 2014).

### 3.4 Radioactive liver injury

Radiation-induced liver injury is a form of non-targeted tissue damage caused by ionizing radiation (such as X-rays and γ-rays) and is primarily characterized by oxidative stress, inflammatory responses, cell apoptosis and fibrosis (Takamatsu et al., 2018). DNA damage is one of the key features of radiation-induced liver injury. When DNA damage exceeds the repair capacity of the cell, it triggers cell cycle arrest or activates signaling pathways such as p53, leading to apoptosis or necrosis (Zhou et al., 2023). Specifically in the liver, DNA damage also promotes proliferation and activation of hepatic stellate cells (HSCs), increasing the risk of fibrosis (Zhou et al., 2023). Currently, antioxidants, anti-inflammatory drugs and anti-fibrotic agents are considered to have potential in alleviating radiation-induced liver injury.

In a γ-ray-induced LO2 cell radiation injury model, AS-IV (20, 40, 80 μg/mL) significantly restored the proliferative capacity of damaged cells, inhibited the increase in ROS and MDA levels, and restored the activities of SOD and GSH (Hu, 2016). Similarly, in a γ-ray-induced mouse radiation injury model, AS-IV (20, 40 mg/kg) significantly ameliorated radiation-induced liver histopathological changes, reduced serum levels of ALT, AST, TNF-α and IL-6, and reversed the activities of MDA and SOD in liver tissue (Yanping et al., 2023). The mechanism may involve AS-IV alleviating radiation-induced liver injury by modulating the Nrf2 and thioredoxin interacting protein (TXNIP)/NLRP3 inflammasome signaling pathways (Hu, 2016; Yanping et al., 2023). These novel findings provide strong theoretical support for the clinical application of AS-IV in the treatment of radiation injury.

### 3.5 Ischemic liver injury

Ischaemia-reperfusion (I/R)-induced liver injury is a complex pathophysiological process commonly encountered in clinical situations such as liver transplantation, liver resection, shock resuscitation and trauma (Wu et al., 2018). When blood flow to the liver is temporarily interrupted for any reason and then restored, the reperfusion process triggers severe oxidative stress, inflammatory responses, endothelial injury, cell apoptosis and autophagy dysregulation, leading to damage to liver tissue structure and function (Jiménez-Castro et al., 2019; Bardallo et al., 2022). In this process, Kupffer cells in the liver play a critical role in the inflammatory response, while neutrophil activation and excessive ROS generation further exacerbate liver injury (Jiménez-Castro et al., 2019). Therefore, the development of effective drugs for the treatment of I/R liver injury is of great importance.

**FIGURE 2:**
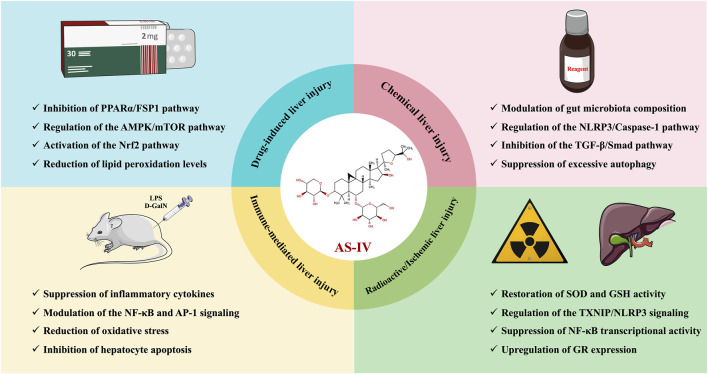
Schematic illustration of the therapeutic effects of AS-IV in liver injury.

In recent years, some plant extracts have received considerable attention for their potential to alleviate I/R liver injury due to their exceptional antioxidant and anti-inflammatory properties. For example, in an *in situ* liver transplantation model of I/R injury, AS-IV significantly improved survival and liver function in mice, while reducing liver parenchymal cell damage (Cheng et al., 2011). Specifically, AS-IV downregulated TNF-α levels, inhibited NF-κB transcriptional activity, and upregulated glucocorticoid receptor expression (Cheng et al., 2011). The protective effect of AS-IV on liver I/R injury may be closely linked to its inhibition of NF-κB transcriptional activity (Cheng et al., 2011). This mechanism could potentially pave the way for the development of novel therapeutic strategies to mitigate I/R injury during liver transplantation. The hepatoprotective effects and mechanisms of AS-IV in liver injury are presented in Table 2, while its therapeutic role in liver injury is schematically illustrated in Figure 2.

**TABLE 2:** The functions and molecular mechanisms of AS-IV in liver injury.

Models	Types	Routes	Dosage of administration	Molecular mechanisms	Years	References
Cisplatin-induced liver injury in C57BL/6 mice	*In vivo*	i.g.	40, 80 mg/kg AS-IV for 9 days	Inhibition of PPARα/FSP1 signaling pathway	2024	Guo et al. (2024)
RSL3-treated AML-12 cells	*In vitro*	N/A	12.5, 25, 50 μM AS-IV for 12 h			
Cisplatin-induced liver and kidney injury in male SD rats	*In vivo*	i.g.	40, 80 mg/kg AS-IV for 7 days	Through autophagy-mediated inhibition of NLRP3	2019	Qu et al. (2019)
APAP-induced liver injury in male ICR mice	*In vivo*	p.o.	20, 40 mg/kg AS-IV for 7 days	Activating Nrf2 antioxidant signaling pathways	2018	Li L. et al. (2018)
STZ-induced diabetic liver injury in SD rats	*In vivo*	i.g.	20, 40, 80 mg/kg AS-IV for 42 days	Upregulating the PI3K/AKT signaling pathway	2017	Xu (2017)
High-sugar + HFD + STZ- induced liver injury in male type 2 diabetic rats	*In vivo*	i.g.	80 mg/kg AS-IV for 56 days	Activating the AMPK/mTOR signaling pathway	2021	Zhu (2021)
CPA-induced oxidative damage and immunosuppression in tilapia	*In vivo*	p.o.	0.1, 0.2, 0.5 g/kg AS-IV for 60 days	Enhance the antioxidant capacity and immune function	2023	Cao et al. (2023)
Ethanol-treated Chang liver cells	*In vitro*	N/A	0.1, 0.5, 1, 5 mg/L AS-IV for 6 h	Regulats the level of ROS, relieves cell cycle arrest in G_0_/G_1_, inhibits apoptosis	2014	Han et al. (2014)
Alcohol-induced liver injury in male Kunming mice	*In vivo*	p.o.	50, 150, 500 mg/kg AS-IV for 7 days	Modulating gut microbiota and regulating NLRP3/Caspase-1 signaling pathway	2023	Wu et al. (2023)
CCl_4_-induced liver fibrosis in male C57BL/6 mice	*In vivo*	i.g.	20, 40 mg/kg AS-IV for 14 days	Decrease TGF-β and Smad3 protein expression	2015	Jiang et al. (2015)
CCl_4_-induced liver injury in male SD rats	*In vivo*	i.g.	40, 80, 160 mg/kg AS-IV for 40 days	Improve oxidative stress and inhibit apoptosis	2019	Xu and Li (2019)
Iron dextran-treated LO2 cells	*In vitro*	N/A	10, 20, 40 μM AS-IV for 24 h	Inhibition of excessive autophagy and apoptosis of iron overload hepatocytes	2019	Xie et al. (2019)
5% sodium taurocholate-induced SAP-associated acute liver injury in male SD rats	*In vivo*	i.p.	20 mg/kg AS-IV for 2 h	Inhibiting JAK2/STAT3 signaling pathway	2016	Zhang and Chang (2016)
LPS-induced acute inflammatory responses in female C57BL/6J mice	*In vivo*	i.p.	10 mg/kg AS-IV for 6 days	Modulation of NF-κB and AP-1 signaling pathways	2015	Zhang and Frei (2015)
D-GalN/LPS-induced acute liver failure in male C57BL6 mice	*In vivo*	i.g.	50, 150, 500 mg/kg AS-IV for 3 days	Regulating the Nrf2/HO-1 and Bcl-2/Bax signaling pathways	2014	Liu (2014)
BCG + LPS-induced immunological liver injury in SD rats	*In vivo*	i.g.	20, 40, 80 mg/kg AS-IV for 10 days	Anti-inflammatory and antioxidant activity	2014	Gong and Wang (2014)
γ-rays irradiated LO2 liver cells	*In vitro*	N/A	20, 40, 80 μg/mL AS-IV for 4–48 h	Activation of Nrf2 signaling pathway	2016	Hu (2016)
Radiation-induced liver inflammation in male Kunming mice	*In vivo*	i.p.	20, 40 mg/kg AS-IV for 30 days	Inhibiting the TXNIP/NLRP3 inflammasome signaling pathway	2023	Yanping et al. (2023)
I/R in male SD murine model of orthotopic liver transplantation	*In vivo*	i.v.	1.5 mL, 100 g/mL AS-IV at 1 h before surger	Inhibiting NF-κB transcriptional activity	2011	Cheng et al. (2011)

## 4 The hepatoprotective effects and mechanisms of AS-IV in MAFLD

Although AS-IV exhibits significant antioxidant and anti-inflammatory effects in various liver injuries, persistent liver damage may lead to metabolic imbalance in the liver, which can subsequently trigger MAFLD. The pathological features of MAFLD include fat accumulation, insulin resistance, and chronic inflammation (Eslam and George, 2021). AS-IV not only repairs early liver injury but also regulates lipid metabolism and improves insulin sensitivity, thereby effectively inhibiting the onset and progression of MAFLD, providing multiple layers of protection for its treatment.

### 4.1 Improvement of insulin and leptin resistance

MAFLD is a major manifestation of metabolic syndrome and is closely associated with leptin and insulin resistance (Gofton et al., 2023). Leptin and insulin are key hormones that regulate energy balance and metabolism in the body (Polyzos et al., 2015). Resistance to both hormones can lead to metabolic disorders, exacerbating lipid abnormalities, chronic inflammation and liver damage (Muzurović et al., 2021). Therefore, improving leptin and insulin resistance is considered an important strategy for the treatment of MAFLD in clinical practice. Interestingly, Wu et al. (2016) showed that AS-IV (25 mg/kg) significantly reduced serum triglyceride and total cholesterol levels, alleviated hepatic lipid accumulation and reduced adipocyte hypertrophy in a high-fat diet (HFD)-induced mouse model. More importantly, AS-IV alleviated leptin resistance by modulating the expression of leptin, leptin receptors and appetite-related genes (Wu et al., 2016). In SH-SY5Y cells, AS-IV was shown to enhance leptin signaling (Wu et al., 2016). Mechanistically, AS-IV may improve lipid metabolism in obese mice by increasing leptin sensitivity and regulating thermogenesis-related networks (Wu et al., 2016).

It is known that overexpression of protein tyrosine phosphatase 1B (PTP1B) increases the transcriptional activity of sterol regulatory element-binding protein 1c (SREBP-1c), leading to hepatic steatosis, reduced hepatic glucose metabolism and hepatic insulin resistance (Teimouri et al., 2022). Interestingly, Zhou et al. (2021) demonstrated that in HepG2 cells treated with insulin or oleic acid, AS-IV (25.6, 51.2, 102.4 μM) significantly inhibited the expression of PTP1B, reduced intracellular triglyceride, total cholesterol and free fatty acid levels and increased glucose consumption. Further studies suggested that AS-IV may improve insulin resistance and lipid accumulation by regulating the PTP1B/IR/IRS-1 and PTP1B/SREBP-1c signaling pathways (Zhou et al., 2021). Furthermore, AS-IV (12.5, 25, 50 mg/kg) demonstrated significant hypoglycaemic effects in an HFD-induced STZ diabetic mouse model, which may be attributed to its inhibition of liver glycogen phosphorylase and glucose-6-phosphatase activities (Lv et al., 2010). These findings further highlight the potential of AS-IV as a therapeutic agent for metabolic diseases.

### 4.2 Reduction of endoplasmic reticulum stress

Endoplasmic reticulum (ER) stress plays a key role in the development and progression of MAFLD. The ER is an essential organelle responsible for protein folding and processing, lipid synthesis and calcium ion storage (Hetz et al., 2015). Under various metabolic stresses, such as oxidative stress and inflammation, ER dysfunction can induce ER stress and activate the unfolded protein response, which in turn affects hepatic lipid metabolism, inflammatory responses and fibrosis (Bettigole and Glimcher, 2015; Lebeaupin et al., 2018). In particular, under conditions of ER stress, the PKR-like endoplasmic reticulum kinase (PERK) and inositol-requiring enzyme 1 (IRE1) pathways in the unfolded protein response can activate SREBP-1c, which in turn upregulates the expression of genes involved in lipid synthesis (Lebeaupin et al., 2018). This leads to an increase in lipid production in hepatocytes and subsequent lipid accumulation.

In FFAs-induced HepG2 cells and primary mouse hepatocytes, AS-IV (50–200 μg/mL) dose-dependently promoted the phosphorylation of AMPK, ACC and SREBP-1c, inhibited the accumulation of mature SREBP-1 and its translocation to the nucleus, thereby reducing the expression levels of lipid synthesis genes such as ACC1, FAS and SCD1 (Zhou et al., 2017). In addition, AS-IV significantly decreased the expression levels of key markers of ER stress, including GRP78, CHOP and p-PERK, in a dose-dependent manner (Zhou et al., 2017). Mechanistically, AS-IV alleviated FFA-induced ER stress and lipid accumulation by activating the AMPK signaling pathway (Zhou et al., 2017). These findings further demonstrate the important role of AS-IV in alleviating ER stress and regulating lipid metabolism, strongly supporting its potential as a therapeutic agent for MAFLD.

### 4.3 Remodeling of intestinal flora

The onset and progression of MAFLD are influenced not only by host metabolism but also by the gut microbiome. Studies have shown that patients with MAFLD often have dysbiosis, characterized by a reduction in beneficial bacteria (such as *Bifidobacterium* and *Lactobacillus*) and an increase in pro-inflammatory and pathogenic strains (such as *Bacteroides* and certain *Clostridia*) (Zhang Y. et al., 2022). Under normal conditions, the gut microbiota plays a critical role in maintaining host health, including maintaining intestinal barrier function, regulating immune responses, and promoting nutrient absorption and metabolic processes (Aron-Wisnewsky et al., 2020). However, poor diet, lifestyle changes and other environmental factors can lead to an imbalance in the structure and composition of the gut microbiota, thereby promoting the development of MAFLD (Leung et al., 2016). This occurs through mechanisms such as compromising intestinal barrier integrity, disrupting bile acid metabolism, reducing short-chain fatty acid production and activating pro-inflammatory immune responses (Leung et al., 2016). Therefore, remodeling the gut microbiota and restoring its balance has been considered a potential therapeutic strategy for MAFLD.

Interestingly, Zhai et al. (2023) systematically investigated the effects of AS-IV on MAFLD using 16S rRNA gene sequencing, bile acid assays, fecal microbiota transplantation and intestinal farnesoid X receptor (FXR) knockout experiments. The results showed that AS-IV (12.5, 25, 50 mg/kg) significantly ameliorated HFD-induced hepatic steatosis and metabolic disturbances (Zhai et al., 2022). This was closely associated with a decrease in the expression of intestinal bile salt hydrolase, which in turn increased the concentration of the intestinal FXR antagonist taurine-β-muricholic acid. In addition, AS-IV inhibited intestinal FXR signaling, reduced intestinal FGF15 expression, thereby reducing liver FXR activation, increased glucagon-like peptide-1 secretion, and ultimately suppressed SREBP-1c-mediated lipogenesis and liver steatosis (Zhai et al., 2022). Overall, AS-IV ameliorated liver steatosis by reshaping the gut microbiota and inhibiting bile acid deconjugation, thereby enhancing liver fat metabolism *via* the intestinal FXR pathway (Zhai et al., 2022). These findings provide new insights into AS-IV as an oral prebiotic for the treatment of MAFLD.

### 4.4 Enhancement of hepatocyte autophagy

Autophagy plays a key regulatory role in the initiation and progression of MAFLD. It is a complex process that is finely regulated by various proteins and signaling pathways and involves multiple steps such as autophagosome formation, maturation, fusion with lysosomes and degradation of intracellular contents (Yim and Mizushima, 2020). Under normal physiological conditions, autophagy prevents excessive lipid accumulation in hepatocytes by degrading lipid droplets and regulating lipid metabolism (Ichimiya et al., 2020). However, under conditions of increased metabolic stress, autophagic function can be inhibited or impaired, leading to excessive lipid accumulation in hepatocytes and contributing to the development of fatty liver disease (Scorletti and Carr, 2022). Moreover, insufficient autophagic activity can induce oxidative stress, ER stress and mitochondrial dysfunction, further activating pro-inflammatory pathways and exacerbating liver inflammation and fibrosis (Ueno and Komatsu, 2017; Allaire et al., 2019). Therefore, targeting autophagic pathways may represent a novel therapeutic strategy for the treatment of MAFLD.

**FIGURE 3:**
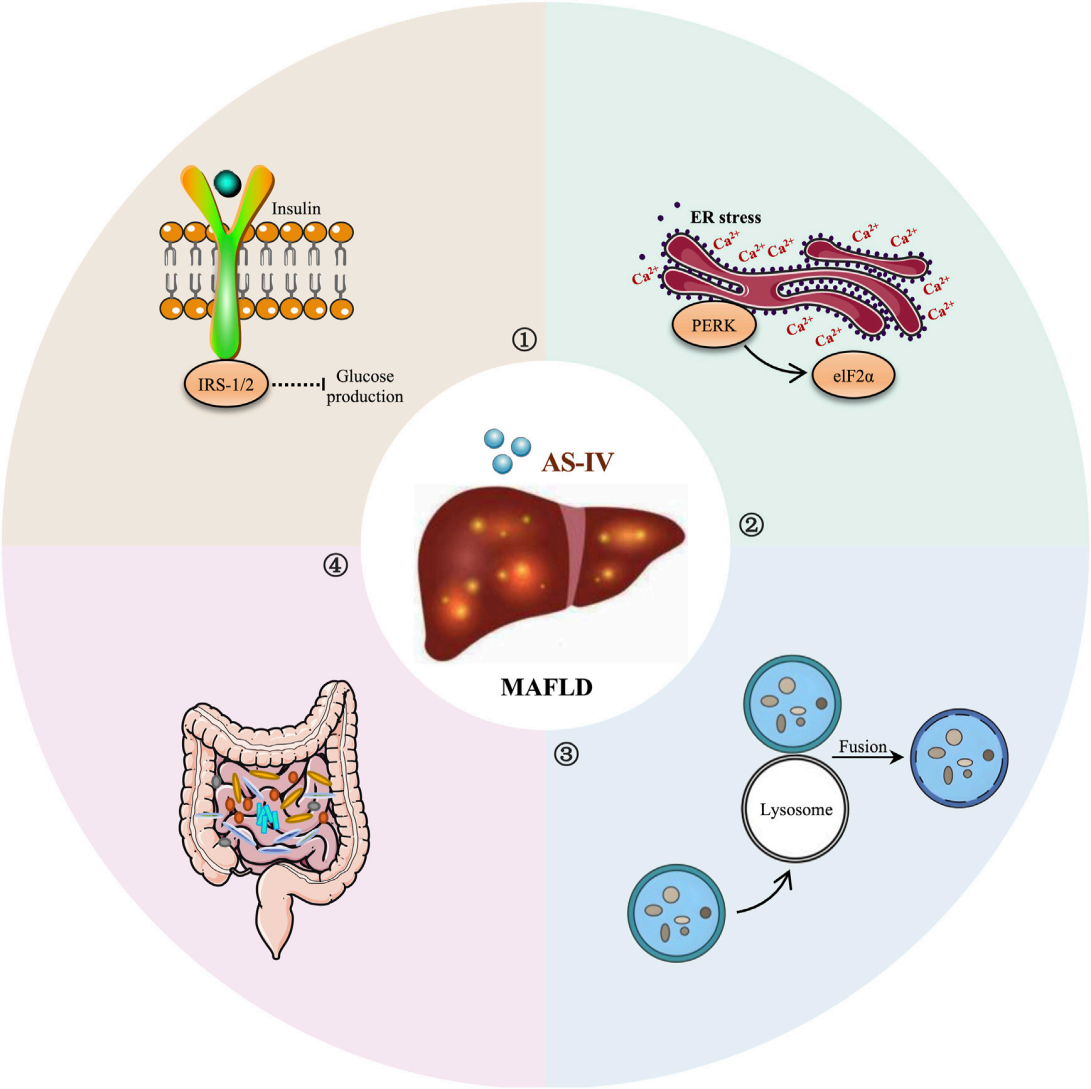
Schematic depiction of the potential of AS-IV in MAFLD treatment.

The regulation of autophagy is known to be closely linked to the Akt/mTOR signaling pathway. Activation of the Akt/mTOR pathway regulates cell growth and metabolism while inhibiting the activity of autophagy-related proteins, thereby affecting cell proliferation, energy metabolism and the pathogenesis of various diseases (Xu et al., 2020). Interestingly, Liu et al. (2024) showed that AS-IV (0.25, 0.5, 1 μg/mL) significantly reduced intracellular lipid accumulation in palmitic acid-induced HepG2 cells. Further experiments showed that AS-IV not only promoted the formation of autophagosomes and increased the expression of the autophagy-related proteins LC3 and Beclin-1, but also reduced the phosphorylation levels of Akt and mTOR and the level of prostacyclin (Liu et al., 2024). Mechanistically, the anti-lipid accumulation effect of AS-IV may enhance hepatocyte autophagy by inhibiting the Akt/mTOR signaling pathway, thereby reducing lipid aggregation (Liu et al., 2024). This finding highlights the potential role of AS-IV in regulating hepatocyte lipid metabolism and autophagic activity, providing new insights for its application in MAFLD. The hepatoprotective effects and mechanisms of AS-IV in MAFLD are summarized in Table 3, while its therapeutic role in MAFLD is depicted schematically in Figure 3.

**TABLE 3:** The functions and molecular mechanisms of AS-IV in MAFLD.

Models	Types	Routes	Dosage of administration	Molecular mechanisms	Years	References
HFD-induced obesity in male C57BL/6 mice	*In vivo*	i.p.	25 mg/kg AS-IV for 13 weeks	Alleviation of leptin resistance and regulation of thermogenic network	2016	Wu et al. (2016)
OA-treated HepG2 cells	*In vitro*	N/A	12.8, 25.6, 51.2, 102.4 μM AS-IV for 24 h	Inhibite PTP1B and effectively improve triglyceride accumulation	2020	Zhou et al. (2021)
HFD + STZ-induced Type 2 diabetes in male C57BL/6J mice	*In vivo*	p.o.	12.5, 25, 50 mg/kg AS-IV for 14 days	Inhibition on hepatic GP and G6Pase activities	2010	Lv et al. (2010)
FFAs-treated human HepG2 cells and primary C57BL/6 murine hepatocytes	*In vitro*	N/A	50–200 μg/mL AS-IV for 24 h	Attenuate ER stress and lipid accumulation in hepatocytes *via* AMPK activation	2017	Zhou et al. (2017)
HFD-induced hepatic steatosis in C57BL/6 male obese mice	*In vivo*	i.g.	12.5, 25, 50 mg/kg AS-IV for 12 weeks	Inhibiting intestinal FXR *via* intestinal flora remodeling	2022	Zhai et al. (2022)
Palmitic acid -treated HepG2 cells	*In vitro*	N/A	0.25–40 μg/mL AS-IV for 24 h	Promote autophagy *via* the Akt/mTOR pathway	2024	Liu et al. (2024)

## 5 The hepatoprotective effects and mechanisms of AS-IV in liver fibrosis

As MAFLD progresses, the accumulation of lipotoxic substances and the sustained chronic inflammatory response in the liver gradually drive the activation of hepatic stellate cells, ultimately leading to liver fibrosis (Hammerich and Tacke, 2023). During this process, AS-IV not only continues its functions in anti-inflammatory and metabolic regulatory functions, but also plays a key role in modulating the TGF-β/Smad signaling pathway. This mechanism contributes to its unique advantage in delaying or even reversing liver fibrosis. As a result, the action of AS-IV shifts from metabolic intervention to the regulation of cellular phenotypes, providing a novel perspective for the treatment of liver fibrosis.

### 5.1 Inhibition of hepatic stellate cell activation

HSCs are the central effector cells in the initiation and progression of liver fibrosis (Higashi et al., 2017). Upon liver injury stimuli, quiescent HSCs are activated into an activated form with myofibroblast characteristics, leading to excessive deposition of extracellular matrix (ECM), particularly collagen, which disrupts liver structure and function (Hammerich and Tacke, 2023). Therefore, inhibition of HSC activation is one of the key strategies for anti-fibrotic therapy. By inhibiting HSC activation, ECM deposition can be reduced, the homeostasis of the liver microenvironment can be restored and the fibrosis process can be reversed (Zhang D. et al., 2022). In particular, key signaling pathways involved in HSC activation, such as TGF-β/Smad and Wnt/β-catenin, are critical targets for current drug development.

In a rat model of porcine serum-induced liver fibrosis, AS-IV (2, 4 mg/kg) significantly reduced serum levels of hyaluronic acid, type III procollagen and TGF-β1, as well as hepatic TGF-β and hydroxyproline content (Liu et al., 2009). Its antifibrotic effect is probably related to the downregulation of TGF-β1, thereby effectively inhibiting HSC activation (Liu et al., 2009). Similarly, in a dimethylnitrosamine-induced rat model of liver fibrosis, AS-IV (10 mg/kg) showed remarkable antifibrotic effects, with the underlying mechanism also possibly related to inhibition of HSC activation (Bai et al., 2020).

In addition, Zhang (Zhang, 2021) showed that AS-IV (20, 40, 80 mg/kg) significantly attenuated DEN-, CCl_4_-and C_2_H_5_OH-induced liver fibrosis in mice by modulating the TGF-β1/p-Smad2/3 signaling pathway. Further investigation revealed that AS-IV also alleviated liver fibrosis induced by these agents by activating the Nrf2/HO-1 signaling pathway (Yang, 2022). In Nrf2 knockout mice, the antifibrotic effect of AS-IV was significantly reduced, highlighting the critical role of the Nrf2 pathway in its mechanism of action (Yang, 2022). More importantly, Nrf2 gene knockout inhibited the antifibrotic effect of AS-IV by downregulating the p-Smad3L/PAI-1 pathway and upregulating the p-Smad3C/p21 pathway (Yang, 2022). These findings further elucidate the antifibrotic potential of AS-IV through the regulation of multiple signaling pathways and provide a theoretical basis for its development as a potential therapeutic agent for liver fibrosis.

### 5.2 Inhibition of oxidative stress

Oxidative stress plays a critical role in the initiation and progression of liver fibrosis, with the primary mechanisms involving excessive ROS generation leading to hepatocyte damage, exacerbated inflammation and activation of HSCs (Sinha et al., 2024). ROS can induce oxidative damage to DNA, proteins and lipids, triggering apoptosis, necrosis and autophagy imbalance, thereby exacerbating the progression of liver fibrosis (Luangmonkong et al., 2018). Moreover, oxidative stress can promote the transformation of HSCs into a fibroblast-like phenotype and accelerate the abnormal deposition of ECM by activating various signaling pathways, such as TGF-β/Smad and mitogen-activated protein kinase (MAPK) pathways (Ramos-Tovar and Muriel, 2020). Hence, inhibition of oxidative stress has become one of the key strategies for anti-fibrotic therapy in liver fibrosis.

Li et al. (2013) showed that AS-IV (1–100 μM) significantly alleviated oxidative stress in activated HSCs. The specific effects were manifested by scavenging ROS, reducing lipid peroxidation levels and increasing intracellular GSH levels by stimulating Nrf2 gene expression, thereby enhancing the antioxidant capacity of the cells (Li et al., 2013). Mechanistically, AS-IV inhibited the generation of oxidative stress and the activation of the p38 MAPK pathway, which further suppressed HSC activation (Li et al., 2013). Similarly, in a rat HSC oxidative damage model induced by H_2_O_2_, AS-IV (100, 200 μM) exhibited significant antioxidant protection and ameliorated oxidative damage. The underlying mechanism may involve the inhibition of Caspase-3 and Caspase-9 expression, thereby suppressing cell apoptosis (Wang et al., 2018). These findings further underscore the potential of AS-IV as a therapeutic agent for liver fibrosis.

### 5.3 Inhibition of inflammatory response

The protease-activated receptor 2 (PAR2) signaling pathway is a key cellular pathway involved in several pathological processes, including liver fibrosis. This pathway exacerbates fibrosis by promoting the secretion of pro-inflammatory factors and activating inflammation-associated immune cells, thereby accelerating both the inflammatory response and fibrosis progression (Shearer et al., 2016). Interestingly, in a CCl_4_-induced rat model of liver fibrosis, AS-IV (120 mg/kg) significantly reduced the secretion of pro-inflammatory cytokines, including IL-1β, IL-6 and TNF-α, while inhibiting the accumulation of collagen markers, including hexadecenoic acid, laminin and hydroxyproline (Wang Z. et al., 2017). Mechanistically, AS-IV alleviated liver fibrosis by suppressing the PAR2 pathway, thereby reducing inflammation and collagen deposition associated with fibrosis (Wang Z. et al., 2017). Notably, this hepatoprotective effect was more pronounced in a mouse model of diabetes-induced liver fibrosis, providing a novel approach for the further development of AS-IV as an anti-fibrosis drug.

TXNIP is known to be an endogenous inflammatory regulator that, when stimulated by danger signals, can bind to NLRP3 to form the TXNIP/NLRP3 complex, thereby activating the NLRP3 inflammasome (Colunga Biancatelli et al., 2022). Unfortunately, excessive activation of the NLRP3 inflammasome is a key mechanism in liver disease, contributing to hepatocyte injury and amplification of inflammation, which significantly exacerbates the progression of liver fibrosis (Szabo and Petrasek, 2015). In a CCl_4_-and ethanol-induced rat model of liver fibrosis, AS-IV (20, 40 mg/kg) significantly attenuated hepatocyte injury and fibrosis and effectively inhibited HSC activation (Su et al., 2023). Mechanistic studies suggest that this anti-fibrotic effect may be related to AS-IV’s inhibition of the TXNIP/NLRP3 inflammasome pathway, which blocks the amplification of inflammatory signals (Su et al., 2023). Furthermore, the study found that AS-IV may further exert its anti-HSC activation and anti-liver fibrosis effects through by regulating the C-X-C motif chemokine ligand 12 (CXCL12)/C-X-C chemokine receptor type 4 (CXCR4) signaling axis (Su et al., 2023). These findings provide important theoretical support for the potential of AS-IV as an anti-fibrotic drug.

### 5.4 Inhibition of epithelial-mesenchymal transition

Epithelial-mesenchymal transition (EMT) is a key pathological process in liver fibrosis, characterized by the loss of polarity and intercellular adhesion properties in hepatic epithelial cells, which then transdifferentiate into mesenchymal cell-like phenotypes with enhanced migration and invasion capabilities (Liu et al., 2022). In liver fibrosis, EMT not only directly promotes abnormal ECM deposition associated with fibrosis, but also exacerbates disease progression by enhancing inflammation and oxidative stress in the fibrotic microenvironment (Yu et al., 2018). Hence, targeting key molecules and signaling pathways involved in the EMT process may provide novel strategies and effective targets for anti-fibrosis therapy. Interestingly, Dai et al. (2022) showed that AS-IV (14 mg/kg) had significant anti-fibrotic effects in a CCl_4_-induced rat liver fibrosis model. The mechanism of action may be closely related to the regulation of key molecules involved in the EMT process (Dai et al., 2022). Specifically, AS-IV was found to downregulate the protein expression of N-cadherin, α-SMA and TGF-β1, while upregulating the expression of E-cadherin, thereby inhibiting the EMT process and alleviating the pathological progression of liver fibrosis (Dai et al., 2022). This finding further highlights the potential of AS-IV in anti-fibrosis therapy and provides new research directions for the development of EMT-related therapeutic targets. The hepatoprotective effects and mechanisms of AS-IV in liver fibrosis are presented in Table 4.

**TABLE 4:** The functions and molecular mechanisms of AS-IV in liver fibrosis.

Models	Types	Routes	Dosage of administration	Molecular mechanisms	Years	References
Porcine serum-induced liver fibrosis in male Wistar rats	*In vivo*	p.o.	2.0, 4.0 mg/kg AS-IV for 12 weeks	Inhibitory effects on collagen synthesis and proliferation	2009	Liu et al. (2009)
DMN-induced hepatic fibrosis in male Wistar rats	*In vivo*	i.g.	10 mg/kg AS-IV for 2 weeks	Inhibiting the activation of HSCs	2020	Bai et al. (2020)
TGF-β1-stimulated HSC-T6 cells	*In vitro*	N/A	5, 10, 20 μmol/L AS-IV for 24 h	Regulating the Smad2/3 link region and C-terminal phosphorylation	2021	Zhang (2021)
DEN/CCl_4_/C_2_H_5_OH- induced liver fibrosis in male C57BL/6J mice	*In vivo*	i.g.	20, 40, 80 mg/kg AS-IV for 12 weeks			
TGF-β1-stimulated HSC-T6 cells	*In vitro*	N/A	5, 10, 20 μM AS-IV for 24 h	Through the Nrf2/HO-1 pathway	2022	Yang (2022)
DEN/CCl_4_/C_2_H_5_OH- induced liver fibrosis in male C57BL/6 mice	*In vivo*	i.g.	20, 40, 80 mg/kg AS-IV for 1-12 weeks			
BSO/SB-203580-treated HSCs	*In vitro*	N/A	1, 3, 10, 30, 100 μM AS-IV for 0–72 h	Inhibiting generation of oxidative stress and associated p38 MAPK activation	2013	Li et al. (2013)
H_2_O_2_-treated rat HSC-T6	*In vitro*	N/A	100, 200 μM AS-IV for 24 h	Inhibition of expressions of Caspase-3 and Caspase-9 to inhibit cells apoptosis	2018	Wang et al. (2018)
STZ + CCl_4_-induced diabetic hepatic fibrosis in male SD rats	*In vivo*	p.o.	120 mg/kg AS-IV for 6 weeks	Inhibiting PAR2 signaling	2017	Wang Z. et al. (2017)
CCl_4_/ethanol-induced liver injury in male SD rats	*In vivo*	i.g.	20, 40 mg/kg AS-IV for 6 weeks	Inhibition of CXCL12/CXCR4 signal axis and TXNIP/NLRP3 inflammatory pathway	2023	Su et al. (2023)
CCl_4_-induced hepatic fibrosis in SD rats	*In vivo*	i.g.	14 mg/kg AS-IV for 4 w	Downregulation of N-cadherin, α-SMA, TGF-β1 protein expression and upregulation of E-cadherin protein expression	2022	Dai et al. (2022)

## 6 The hepatoprotective effects and mechanisms of AS-IV in liver cancer

During the process of liver fibrosis, the continuous remodeling of the extracellular matrix and abnormal tissue repair create a “precancerous microenvironment” that facilitates malignant transformation of hepatocytes (Vogel et al., 2022). Notably, the microenvironmental regulatory network formed by AS-IV in the anti-fibrosis process complements its antitumor mechanisms in liver cancer prevention and treatment. By inhibiting EMT, inducing tumor cell apoptosis, and suppressing abnormal angiogenesis, AS-IV demonstrates its multidimensional regulatory properties throughout the progression of liver disease, making it a highly promising candidate for sequential therapy in liver diseases.

### 6.1 Inhibition of proliferation of HCC cells

Inhibiting the proliferation of liver cancer cells is a crucial strategy in the treatment of HCC, as the rapid proliferation of cancer cells is a hallmark of HCC progression and deterioration (Anwanwan et al., 2020). By blocking the proliferation of liver cancer cells, tumour growth and metastasis can be effectively controlled, thereby delaying HCC progression and improving patient prognosis. A large body of research indicates that AS-IV can effectively inhibit the proliferation of various liver cancer cell lines, including SNU-182, Huh-7, HepG2, SK-Hep1, Hep3B, SMMC-7721 and Bel-7402 cells (Qi et al., 2010; Qin et al., 2017; Su et al., 2020; Guo et al., 2022; Zhu and Lu, 2024). Similarly, Li G. et al. (2017) and Cui et al. (2020) confirmed that AS-IV significantly inhibited the proliferation of SMMC-7721 and Huh-7 cells, and this inhibitory effect was both concentration- and time-dependent.

Lysine acetyltransferase 2A (KAT2A) is a histone acetyltransferase that primarily regulates gene expression by catalyzing the transfer of acetyl groups (Li et al., 2023). Phosphoglycerate mutase 1 (PGAM1), a key enzyme in the glycolytic pathway, is closely associated with tumour cell proliferation and survival (Yang et al., 2022). Recent studies have shown that KAT2A-mediated PGAM1 succinylation plays a critical role in promoting metabolic reprogramming of cancer cells and enhancing their antioxidant capacity. This mechanism may provide new potential therapeutic targets for cancer treatment. Interestingly, Zhu et al. (Li Y. et al., 2018) showed that AS-IV (20 μg/mL) significantly reduced the viability, glucose consumption, lactate production, extracellular acidification rate and succinylation levels of SNU-182 and Huh7 cells, whereas KAT2A overexpression reversed these effects. Mechanistically, AS-IV inhibited liver cancer cell viability and glycolysis by regulating KAT2A-mediated PGAM1 succinylation (Li Y. et al., 2018). In addition, AS-IV (150 μg/mL) downregulated the expression of Vav3.1 in HepG2 cells in a dose- and time-dependent manner, exerting anti-liver cancer effects (Qi et al., 2010). Notably, the downregulation of Vav3.1 was closely associated with the inhibition of malignant transformation (Boesch et al., 2018).

### 6.2 Inhibition of invasion and migration of HCC cells

Inhibiting the ability of liver cancer cells to invade and migrate is a critical goal in liver cancer therapy, as the high invasiveness and metastatic potential of liver cancer are key factors contributing to poor patient prognosis (Yüksel et al., 2017). Preventing or slowing the invasion and migration of liver cancer cells can effectively limit the spread of cancer to other organs, thereby improving treatment outcomes. The activation of EMT is considered a critical event in the early stages of tumour metastasis (Puisieux et al., 2014). He et al. (2020) showed that AS-IV (25, 50, 100 nM) can inhibit the invasion and migration of HepG2 cells by regulating the EMT process. Specifically, after treatment with AS-IV, the expression level of E-cadherin was significantly increased, while the expression levels of N-cadherin and vimentin were significantly decreased (He et al., 2020). This inhibitory effect may be related to modulation of the Wnt/β-catenin signaling pathway (He et al., 2020). Similarly, Qin et al. (2017) confirmed that AS-IV (10, 50, 100 μg/mL) could dose-dependently suppress EMT, thereby attenuating the invasive and migratory abilities of liver cancer cells. The mechanism may involve inhibition of EMT through regulation of the Akt/GSK-3β/β-catenin signaling pathway (Qin et al., 2017).

In addition, AS-IV (5, 10, 20 μM) can inhibit the proliferation, migration and invasion of Huh-7 cells (Li L. et al., 2022). Further studies suggested that this effect may be related to the activation of the Nrf2/HO-1 pathway, which promotes the tumour suppressive effects mediated by pSmad3C/p21, while inhibiting the pro-cancer effects of pSmad3L/c-Myc (Li L. et al., 2022). Based on bioinformatics analysis, Zhou (Zhou, 2022) investigated the core targets and key signaling pathways of AS-IV in HCC. More importantly, combined with *in vitro* experiments, the study systematically investigated the effects of AS-IV on proliferation, migration, invasion, cell cycle, apoptosis, and the expression of key genes and proteins in HepG2 and Huh7 cells (Zhou, 2022). The results showed that AS-IV (45, 90, 180 μg/mL) significantly inhibited the migration, invasion and proliferation of liver cancer cells and promoted their apoptosis (Zhou, 2022). This mechanism of action may be related to the regulation of vascular endothelial growth factor A (VEGFA) and TGF-β1 expression by AS-IV through multiple targets and pathways (Zhou, 2022).

A large body of research has confirmed that lncRNAs are dysregulated in various cancers, suggesting their critical role in cancer initiation and progression (Li J. K. et al., 2017; Yuan et al., 2017). Among them, lncRNA-ATB is recognized as an oncogenic lncRNA that promotes EMT and metastasis in HCC cells by competitively binding to the miR-200 family (Yuan et al., 2014). Li Y. et al. (2018) showed that AS-IV (160 μg/mL) significantly downregulated the expression of lncRNA-ATB in HCC cells in a dose- and time-dependent manner. Further investigation revealed that overexpression of lncRNA-ATB could reverse the effects of AS-IV on HCC cell migration, EMT, apoptosis, cell viability and IL-11/STAT3 signaling pathway (Li Y. et al., 2018). These effects were likely due to the inhibition of lncRNA-ATB by AS-IV, which in turn suppressed HCC cell migration and viability (Li Y. et al., 2018).

### 6.3 Induction of apoptosis of HCC cells

Liver cancer cells are known to be typically resistant to apoptotic signals, leading to their rapid proliferation and spread throughout the body (Singh and Lim, 2022). By activating apoptotic signaling pathways, cancer cells can undergo programmed cell death, thereby reducing the number of tumour cells and inhibiting their invasion and metastatic potential (Anwanwan et al., 2020). Notably, common apoptotic induction mechanisms include the activation of both intrinsic and extrinsic pathways, as well as the regulation of Bcl-2 family proteins and Caspase family proteins (Chen H. J. et al., 2023). Therefore, targeting apoptotic pathways offers a potential therapeutic advantage in liver cancer, particularly in the treatment of refractory and advanced stages. Interestingly, Su et al. (2020) found that AS-IV (200, 400 μM) induced G1 phase arrest and apoptosis in SK-Hep1 and Hep3B cells. Specifically, AS-IV significantly increased the cleavage and activation of extrinsic apoptosis-dependent Caspase-8 and intrinsic apoptosis-dependent Caspase-9 (Su et al., 2020). In addition, AS-IV dose-dependently reduced the expression of anti-apoptotic proteins, including X-linked inhibitor of apoptosis protein, myeloid cell leukemia-1, cellular flice-like inhibitory protein and surviving (Su et al., 2020). Taken together, AS-IV not only induces apoptosis and G1 arrest through both intrinsic and extrinsic pathways, but also attenuates the invasive ability and anti-apoptotic mechanisms of liver cancer cells (Su et al., 2020).

MicroRNAs (miRNAs) are a class of small non-coding RNAs that regulate gene expression post-transcriptionally by binding to specific mRNAs (Bartel, 2018). Recent studies have shown that miRNAs play a critical role in the initiation, progression and metastasis of liver cancer (Liang et al., 2020). Their specific mechanisms include the regulation of proliferation, migration, invasion and apoptosis of liver cancer cells (Khare et al., 2022). For example, miR-150-5p inhibits tumourigenesis and metastasis in HCC cells by targeting c-Myb (Lan et al., 2019). Therefore, miRNA-based targeted therapy is emerging as a promising strategy for the treatment of liver cancer. Cui et al. (2020) showed that AS-IV (5–80 μg/mL) significantly upregulated miR-150-5p expression and decreased β-catenin levels. Mechanistically, AS-IV may promote apoptosis by regulating the miR-150-5p/β-catenin axis, thereby inhibiting the progression of HCC (Cui et al., 2020). These findings provide new insights into the potential use of AS-IV in the treatment of liver cancer.

### 6.4 Inhibition of angiogenesis of HCC cells

Angiogenesis is a critical process for tumour growth and metastasis, providing oxygen and nutrients to support rapid cancer cell proliferation (Morse et al., 2019). Given the high vascular dependency of HCC, inhibition of angiogenesis has become an effective strategy to limit tumour growth and metastasis (Hu et al., 2022). Typically, angiogenesis inhibition is achieved by targeting VEGF and its associated signaling pathways such as VEGFR, PDGFR and FGFR (Ghalehbandi et al., 2023). Zhang et al. (2017) showed that AS-IV (20 mg/kg) significantly inhibited tumour growth and angiogenesis in an orthotopic nude mouse model of HCC. This effect was characterized by a reduction in the expression of angiogenesis-related factors, including VEGF, FGF2 and matrix metalloproteinase 2 (MMP2), as well as the thrombotic factors TF and FVII (Zhang et al., 2017). The anti-angiogenic mechanism of AS-IV may be related to the upregulation of miR-122 and downregulation of miR-221 (Zhang et al., 2017). In addition, Zhou (Zhou, 2022) identified VEGFA as a core gene in HCC, with the VEGF signaling pathway being the most enriched pathway. *In vitro* experiments demonstrated that AS-IV significantly reduced VEGFA protein expression in HepG2 and Huh-7 cells (Zhou, 2022). These results suggest that AS-IV may exert its anti-tumour effects by regulating the VEGF pathway, inhibiting the synthesis of angiogenesis-related factors, and suppressing the proliferation and migration of liver cancer cells (Zhou, 2022).

### 6.5 Enhancement of sensitivity to anti-cancer drugs

Multidrug resistance (MDR) is the phenomenon whereby tumour cells develop resistance to multiple chemotherapeutic agents and remains one of the major challenges in cancer treatment (Mullard, 2020). The development of MDR involves several mechanisms, including overexpression of drug efflux pumps (such as P-gp, MRP1 and BCRP), alterations in apoptotic pathways and increased DNA repair capacity, which reduce drug accumulation in tumour cells or diminish their cytotoxic effects (Madukwe, 2023). Encouragingly, numerous studies have shown that AS-IV, as an effective MDR reversal agent, can increase the sensitivity of tumour cells to chemotherapeutic drugs, thereby synergistically enhancing anticancer effects (Wang et al., 2014). For example, AS-IV (0.08 mg/mL) can reverse the drug resistance of Bel-7402/FU cells by downregulating the expression of MDR1 (Wang et al., 2014; Wang P. P. et al., 2017; Qu et al., 2020). Further research showed that AS-IV (0.1 mM) reversed the drug resistance of Bel-7402/FU cells by inhibiting the JNK/c-Jun/AP-1 signaling pathway and downregulating the expression of MDR1 (Wang P. P. et al., 2017). However, it remains to be investigated whether other MAPK signaling pathways (such as ERK and p38 MAPK) are involved in AS-IV-induced MDR1 downregulation. In addition, cisplatin-induced overexpression of MRP2 is an important mechanism that reduces chemotherapy sensitivity and leads to resistance (Rocha et al., 2018). Qu et al. (2020) showed that AS-IV (0.4, 4, 40 μM) enhanced the antitumour activity of cisplatin in HCC cells by inhibiting MRP2 expression. These findings provide new insights into the combined use of chemotherapeutic drugs and natural metabolites in the treatment of liver cancer.

### 6.6 Others

**FIGURE 4:**
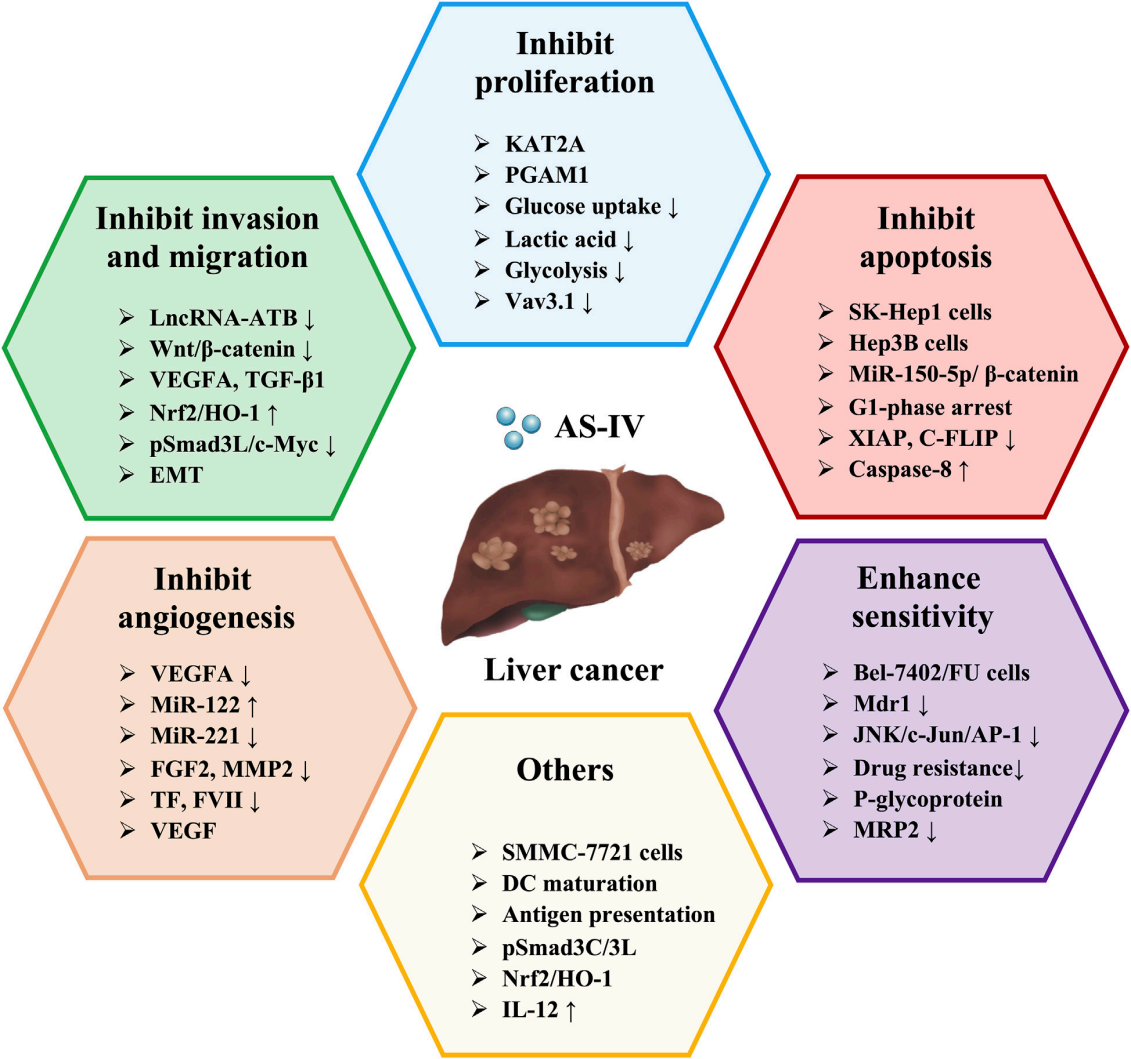
Schematic representation of the role of AS-IV in liver cancer.

Existing studies suggest that enhancing the ability of dendritic cells (DCs) to recognize tumour antigens or upregulating the expression of costimulatory molecules can effectively activate anti-tumour immune responses (Chen et al., 2024). Therefore, DC-based immunotherapy holds great promise for inhibiting tumour progression. Interestingly, Guo et al. (2022) investigated the regulatory effects of AS-IV on DC-mediated antitumour immunity and explored its potential as a novel immune adjuvant. The results showed that AS-IV promoted DC maturation and antigen-presenting function, significantly increased IL-12 secretion and enhanced DC-induced specific cytotoxic responses against SMMC-7721 cells (Guo et al., 2022). This suggests that AS-IV may serve as a natural immune adjuvant for DC-mediated anti-tumour therapy in liver cancer. Furthermore, Zhang et al. (2021) validated the inhibitory effects of AS-IV on DEN/CCl_4_/C_2_H_5_OH-induced liver cancer in mice and on the proliferation, migration and invasion of TGF-β1-stimulated Huh-7 cells through both *in vitro* and *in vivo* experiments. Mechanistically, the anticancer effects of AS-IV may be related to the regulation of the pSmad3C/L and Nrf2/HO-1 signaling pathways (Zhang et al., 2021). The hepatoprotective effects and mechanisms of AS-IV in liver cancer are summarized in Table 5, while the schematic representation of the effects of AS-IV in liver cancer is shown in Figure 4.

**TABLE 5:** The functions and molecular mechanisms of AS-IV in liver cancer.

Models	Types	Routes	Dosage of administration	Molecular mechanisms	Years	References
PHHs, SNU-182 and Huh7 cells	*In vitro*	N/A	0–40 μg/mL AS-IV for 12 h	Regulating KAT2A-mediated succinylation of PGAM1	2024	Zhu and Lu (2024)
Male BALB/c mice + Huh7 cells	*In vivo*	p.o.	40 mg/kg AS-IV for 4 weeks			
HepG2 cells	*In vitro*	N/A	0–200 μg/mL AS-IV for 24 h-14 days	Suppression of Vav3.1 expression	2010	Qi et al. (2010)
SK-Hep1 and Hep3B cells	*In vitro*	N/A	0–400 μM AS-IV for 0–48 h	Induce apoptosis, G_1_-phase arrest, diminishes cell invasion and inhibit anti-apoptotic signaling	2020	Su et al. (2020)
SMMC-7721 cells	*In vitro*	N/A	25–200 mg/mL AS-IV for 1–7 days	Enhance anti-hepatocarcinoma immunity of dendritic cells	2022	Guo et al. (2022)
Huh7 and MHCC97-H cells	*In vitro*	N/A	0–100 μg/L AS-IV for 24 h-14 days	Inhibits metastasis through the suppression of EMT via the Akt/GSK-3β/β-catenin pathway	2017	Qin et al. (2017)
SMMC-7721 and Huh7 cells	*In vitro*	N/A	0–200 μg/mL AS-IV for 0–72 h	Regulating miR-150-5p/β-catenin axis	2020	Cui et al. (2020)
Male BALB/c nude mice+ SMMC-7721 cells	*In vivo*	N/A	20 μg/mL AS-IV for 15 days			
SMMC-7721 and huh-7 cells	*In vitro*	N/A	0–160 μg/mL AS-IV for 0–72 h	Through decreasing lncRNA-ATB expression	2018	Li L. et al. (2018)
HepG2 cells	*In vitro*	N/A	25, 50, 100 nM AS-IV for 24 h	Regulating Wnt/β-catenin pathway	2020	He et al. (2020)
Huh-7 cells	*In vitro*	N/A	5, 10, 20 μM AS-IV for 0–24 h	Activation of the Nrf2/HO-1 pathway, upregulation of the pSmad3C/p21 pathway, and downregulation of the pSmad3L/c-Myc pathway	2022	Li L.et al. (2022)
HepG2 and Huh7 cells	*In vitro*	N/A	0–180 μg/mL AS-IV for 24–72 h	Regulation of VEGFA and TGF-β1 expression through multi-target and multi-pathway	2022	Zhou (2022)
Male BALB/c nude mice + HepG2 cells	*In vivo*	p.o.	20 mg/kg AS-IV for 21 days	Upregulation of miR-122 and downregulation of miR-221	2017	Zhang et al. (2017)
Bel-7402 and Bel-7402/FU cells	*In vitro*	N/A	0.04–0.64 mg/mL AS-IV for 48 h	Reduces the expression level of P-glycoprotein	2014	Wang et al. (2014)
Bel-7402/FU cells	*In vitro*	N/A	0.1 mM AS-IV for 24 h	Inhibiting the JNK/c-Jun/AP-1 signaling pathway	2017	Wang P. P.et al. (2017)
HepG2 cells	*In vitro*	N/A	0.4–40 μM AS-IV for 24 h	Enhance cisplatin chemosensitivity by suppressing MRP2 expression	2020	Qu et al. (2020)
Male BALB/c mice + H22 cells	*In vivo*	p.o.	50 mg/kg AS-IV for 14 days			
DEN/CCl_4_/C_2_H_5_OH-induced primary liver cancer in male C57BL/6J mice	*In vivo*	i.g.	20, 40, 80 mg/kg AS-IV for 20 weeks	Regulating the pSmad3C/3L and Nrf2/HO-1 pathways	2021	Zhang et al. (2021)
TGF-β_1_-treated HSC-T6/HepG2 cells	*In vitro*	N/A	5, 10, 20 μM AS-IV for 24 h			

## 7 Toxicity of AS-IV

Current evidence indicates that AS-IV has a high safety profile, with no studies reporting lethal toxicity. Long-term toxicological studies also suggest that AS-IV has low toxicity. For instance, after 14 weeks of oral administration of AS-IV (2.5, 5, 10 mg/kg) in rats, no significant adverse effects were observed (Gui et al., 2012). Furthermore, in a clinical trial involving healthy Chinese volunteers, single intravenous doses of 200–600 mL and multiple daily doses of 500 mL over 1 week (0.09 mg/mL) did not result in any toxic reactions or plasma accumulation (Xu et al., 2013). However, it is important to note that AS-IV may affect fetal development. Studies have shown that intravenous administration of AS-IV (1.0 mg/kg) to pregnant rats resulted in delayed fur development, prolonged eye-opening time, and slower cliff avoidance reflexes in their offspring (Xuying et al., 2010). Interestingly, no significant changes in learning and memory abilities were observed (Xuying et al., 2010). This suggests that pregnant women should exercise caution when using AS-IV to avoid potential risks.

Overall, AS-IV demonstrates extremely low toxicity and exhibits a high level of clinical safety, indicating its broad potential for clinical application. However, further systematic toxicological studies are needed to comprehensively evaluate the toxicity, potential side effects, and mechanisms of action of AS-IV, providing more robust scientific evidence for its widespread clinical use.

## 8 Discussion and future perspective

**FIGURE 5:**
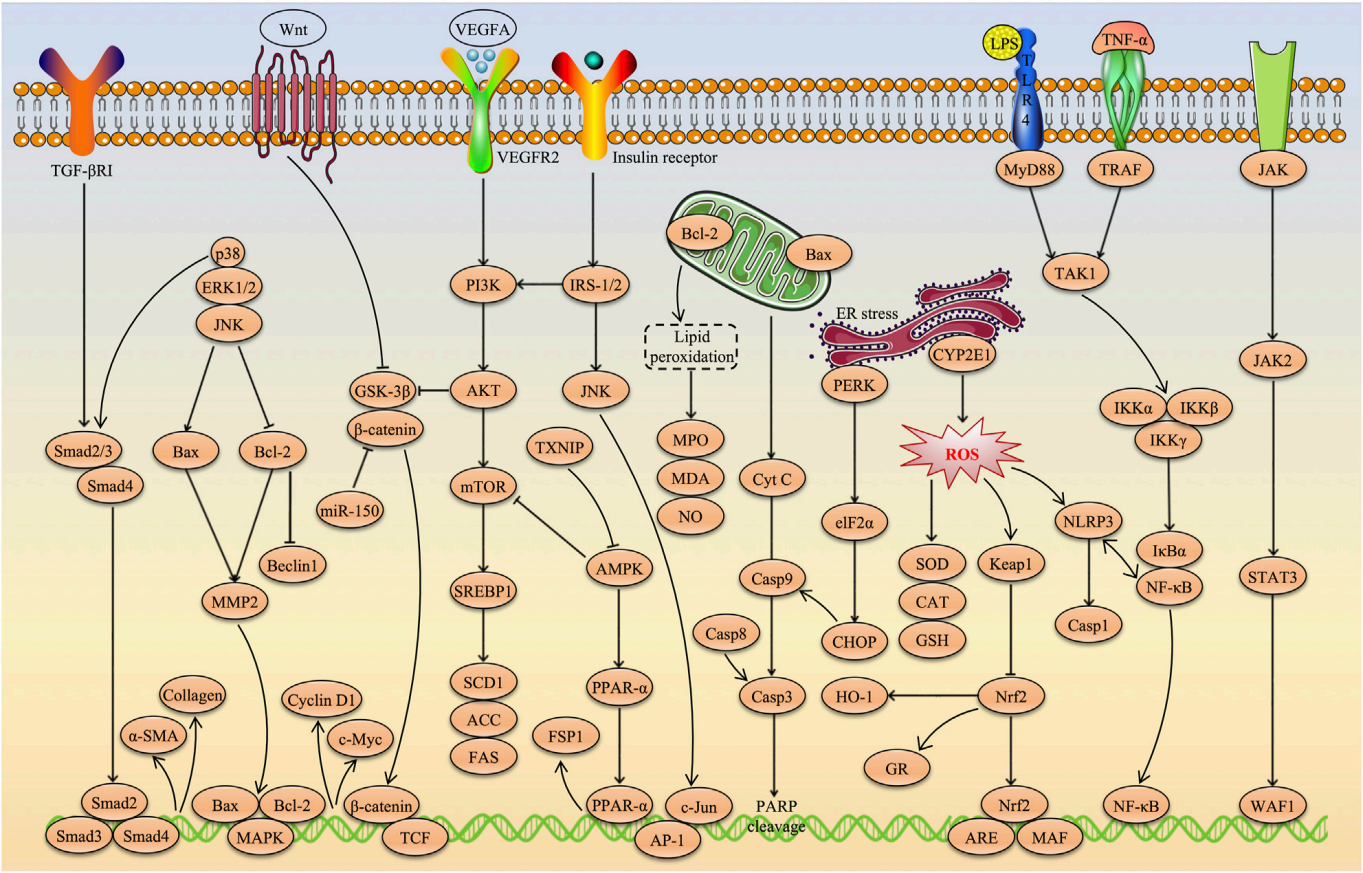
Molecular pathways underlying the hepatoprotective effects of AS-IV.

AS-IV, a natural triterpenoid saponin metabolite, has recently attracted considerable attention in academic research due to its remarkable hepatoprotective effects. A growing body of evidence suggests that AS-IV has great potential in the treatment of various liver diseases, including multi-causal liver injury, MAFLD, liver fibrosis and HCC. The hepatoprotective effects of AS-IV involve multiple molecular mechanisms, such as alleviating oxidative stress and inflammatory responses, ameliorating insulin and leptin resistance, inhibiting excessive autophagy and ER stress, suppressing angiogenesis, remodeling the gut microbiota, preventing ferroptosis, and inhibiting HCC cell proliferation, invasion and migration while promoting tumour cell apoptosis. More importantly, key signaling pathways such as Nrf2/HO-1, PI3K/AKT/mTOR, NF-κB, TGF-β1/p-Smad2/3, TXNIP/NLRP3, JNK/c-Jun/AP-1, CXCL12/CXCR4, PPARα/FSP1, PTP1B/SREBP-1c and Wnt/β-catenin were identified as critical targets for the hepatoprotective effects of AS-IV. These findings not only provide strong scientific evidence supporting AS-IV as a potential therapeutic agent for liver diseases, but also offer a theoretical foundation for further evaluation of its clinical applicability. The molecular pathways underlying the hepatoprotective effects of AS-IV are illustrated in Figure 5.

Despite a growing body of research demonstrating the broad therapeutic effects of AS-IV in liver disease, including antioxidant, anti-inflammatory, anti-fibrotic and modulation of the gut-liver axis, its clinical application still faces significant challenges. Currently, the standard of care for liver disease includes synthetic drugs such as ursodeoxycholic acid (UDCA) and obeticholic acid (OCA), as well as emerging biological therapies such as FGF21 agonists and GLP-1 receptor agonists. UDCA and OCA are primarily used to treat cholestatic liver diseases, with OCA working by activating the FXR to regulate bile acid metabolism, reduce inflammation, and fibrosis (Farooqui et al., 2022; Levy et al., 2023). However, OCA can lead to side effects such as pruritus and elevated low density lipoprotein levels (Manne and Kowdley, 2019). Existing studies suggest that AS-IV may also improve bile acid metabolism *via* the FXR pathway and has shown promising safety in animal studies (Zhai et al., 2022). Hence, AS-IV holds potential as a candidate for modulating bile acid metabolism. However, its potency and clinical efficacy still require further investigation.

In recent years, FGF21 agonists and GLP-1 receptor agonists have shown promising efficacy in MASLD, exerting their effects by improving insulin resistance, reducing fat accumulation, and alleviating inflammation (Mantovani and Dalbeni, 2021; Wei et al., 2024). Similarly, AS-IV has demonstrated the ability to regulate lipid metabolism, enhance insulin sensitivity, and possess anti-inflammatory properties in animal studies (Wu et al., 2016; Li L. et al., 2018; Zhu, 2021). However, there is currently a lack of direct comparative studies regarding the onset time, potency, and long-term efficacy of AS-IV in relation to these agonists. Therefore, future research could further evaluate the feasibility of combining AS-IV with FXR agonists, GLP-1 receptor agonists, and other therapies to optimize treatment regimens, improve efficacy, and reduce adverse effects associated with monotherapy.

However, there are some discrepancies in the reported effects of AS-IV across different studies, which may be attributed to factors such as experimental design, individual animal variability, or dosing differences. For example, the choice of animal model used in experiments may influence the pharmacological effects of AS-IV, as models such as CCl_4_-induced, DEN-induced, C_2_H_5_OH-induced and HFD-induced models exhibit significant differences in inflammation, fibrosis, and metabolic characteristics. In addition, differences in species, age, and gut microbiota composition in experimental animals could impact the effects of AS-IV and its metabolic pathways. Dose-dependent effects are also a crucial factor, as variations in AS-IV dosing and routes of administration (e.g., intraperitoneal injection, oral gavage, or dietary supplementation) may lead to changes in pharmacokinetic properties, influencing its hepatic accumulation and therapeutic outcomes. Therefore, future research should aim to standardize experimental conditions, optimize the dose-response relationship, and integrate multi-omics approaches to further elucidate the mechanisms of AS-IV action, ensuring the reproducibility of results and the reliability of clinical translation. Overall, while AS-IV holds great potential in the treatment of liver diseases, its mechanisms of action, pharmacokinetic properties, and clinical efficacy still require further investigation compared to current standard therapies.

Therefore, in order to promote the clinical translational application of AS-IV in the treatment of liver diseases, we propose the following research directions and optimization strategies.

### 8.1 Identify targets and synergistic mechanisms of multiple pathways

Although existing studies have confirmed that multiple signaling pathways are involved in the hepatoprotective effects of AS-IV, the interactions and synergistic mechanisms between these pathways remain incompletely understood, limiting the systematic understanding of the underlying mechanisms. Future research should focus on emerging areas such as the gut-liver axis, ferroptosis and hepatocyte metabolic reprogramming to explore the precise mechanisms by which AS-IV exerts its effects, identify key targets and establish multi-pathway regulatory networks. Moreover, the application of advanced multi-omics technologies-including metagenomics, metabolomics, proteomics, and single-cell sequencing-coupled with integrative bioinformatics analysis, will facilitate a comprehensive understanding of the regulatory patterns of AS-IV within complex biological networks and elucidate its multi-dimensional biological effects.

### 8.2 Enhance pharmacokinetics and safety evaluation studies

Current research on the pharmacokinetic properties, tissue distribution and metabolism processes of AS-IV remains insufficiently systematic and comprehensive, limiting its further development and clinical application. Therefore, it is crucial to strengthen systematic pharmacokinetic studies to thoroughly elucidate the absorption, distribution, metabolism and excretion of AS-IV, to clarify its pharmacokinetic-pharmacodynamic relationship, and to characterize its dose-dependent features in order to optimize therapeutic dosing and administration regimens. In addition, priority should be given to long-term toxicology and safety evaluation studies, with a particular emphasis on patients with liver disease, to assess the tolerability, potential adverse effects, and drug-drug interactions of AS-IV. These efforts will provide robust scientific evidence and safety assurances for its clinical translation and application.

### 8.3 linical research based on disease models and personalized application

Currently, most studies on the hepatoprotective effects of AS-IV have focused on *in vitro* experiments and animal models, and there is a lack of systematic clinical research verification. Therefore, future efforts should prioritize multi-center clinical trials, especially in different types of liver diseases (such as MAFLD, liver fibrosis and liver cancer), to evaluate its specific efficacy and mechanisms of action. In addition, the concept of precision medicine should be integrated to explore the potential for personalized treatment with AS-IV in different populations, to identify relevant biomarkers and to promote the development and clinical application of personalized medication strategies. This will provide stronger scientific support and a basis for the clinical translation of AS-IV.

### 8.4 Development of novel drug delivery systems

Due to the poor water solubility and limited bioavailability of AS-IV, its clinical efficacy is significantly restricted. The development of innovative drug delivery systems has become a key strategy to overcome these challenges. Future efforts should focus on the use of nanocarrier technology, targeted delivery systems and sustained release formulations to enhance the stability of AS-IV *in vivo*, improve its solubility and membrane permeability, thereby increasing its bioavailability and therapeutic efficacy. Furthermore, the integration of multifunctional drug delivery platforms could enable controlled drug release and further optimize its pharmacokinetic profile. These advanced delivery systems are expected to improve the clinical outcomes of AS-IV and promote its widespread use in the prevention and treatment of liver disease.

### 8.5 Exploring combined therapeutic strategies

To enhance the clinical efficacy and expand the therapeutic potential of AS-IV, it is important to explore combination therapy strategies with existing treatments for liver disease. Given its antioxidant, anti-inflammatory, and anti-fibrotic properties, AS-IV shows potential for synergistic effects when combined with current therapeutic agents, such as nucleoside analogs, bile acid derivatives, and anti-fibrotic drugs, thereby improving overall treatment outcomes. Combining therapies not only has the potential to improve efficacy and broaden indications but also helps to reduce drug resistance and adverse effects associated with long-term use of a single medication. Additionally, AS-IV may be considered as an adjunctive therapy in combination with newer drugs such as GLP-1 receptor agonists or SGLT2 inhibitors, which are increasingly being explored in the treatment of metabolic liver diseases. Future research should focus on elucidating the potential mechanisms of drug interactions, optimizing dose ratios, and determining the ideal treatment regimens, providing a more precise therapeutic foundation for clinical application.

In conclusion, this paper systematically summarizes the source, physicochemical properties, toxicity, hepatoprotective pharmacological effects, and potential molecular mechanisms of AS-IV, while thoroughly analyzing the current research gaps and future development directions. By organizing and synthesizing relevant findings, this review enhances the understanding of the hepatoprotective effects of AS-IV, provides a solid theoretical foundation for its mechanistic research and clinical application, and offers valuable references for future exploration and development.

## Glossary

AS-IV: Astragaloside IV
MAFLD: metabolic-associated fatty liver disease
HCC: hepatocellular carcinoma
Nrf2: nuclear factor erythroid 2-related factor 2
HO-1: heme oxygenase 1
NF-κB: nuclear factor kappa
NLRP3: NOD-like receptor family pyrin domain containing 3
JNK: c-Jun N-terminal kinase
AP-1: activator protein 1
PPARα: peroxisome proliferator-activated receptor alpha
FSP1: fibroblast specific protein 1
Akt: protein kinase B
GSK-3β: glycogen synthase kinase 3β
APAP: acetaminophen
GSH: glutathione
ALT: alanine aminotransferase
AST: aspartate aminotransferase
SOD: superoxide dismutase
IL-1β/6: interleukin-1β/6
TNF-α: tumor necrosis factor α
STZ: streptozotocin
PI3K: phosphatidylinositide 3-kinase
AMPK: AMP-activated protein kinase
mTOR: mammalian target of rapamycin
CPA: cyclophosphamide
ROS: reactive oxygen species
CAT: catalase
MDA: malondialdehyde
LPS: lipopolysaccharide
LBP: LPS-binding protein
DAO: diamine oxidase
MPO: myeloperoxidase
CCl4: carbon tetrachloride
Smad3: mothers against decapentaplegic homolog 3
JAK2: Janus kinase 2
STAT3: signal transducer and activator of transcription 3
MCP-1: monocyte chemoattractant protein-1
D-GalN: D-galactosamine
Bcl-2: B-cell lymphoma-2
Bax: Bcl-2-associated X
BCG: *Bacillus* Calmette-Guérin
HSCs: hepatic stellate cells
TXNIP: thioredoxin interacting protein
I/R: Ischemia-reperfusion
HFD: high-fat diet
PTP1B: protein tyrosine phosphatase 1B
SREBP-1c: sterol regulatory element-binding protein 1c
ER: endoplasmic reticulum
PERK: PKR-like endoplasmic reticulum kinase
IRE1: inositol-requiring enzyme 1
FXR: farnesoid X receptor
ECM: extracellular matrix
MAPK: mitogen-activated protein kinases
PAR2: protease-activated receptor 2
CXCL12: C-X-C motif chemokine ligand 12
CXCR4: C-X-C chemokine receptor type 4
VEGFA: vascular endothelial growth factor A
EMT: epithelial-mesenchymal transition
KAT2A: lysine acetyltransferase 2A
PGAM1: phosphoglycerate mutase 1
miRNAs: microRNAs
MMP2: matrix metalloproteinase 2
MDR: multidrug resistance
DCs: dendritic cells
UDCA: ursodeoxycholic acid
OCA: obeticholic acid.
